# Supplementary material for: HIF sustain a transcriptional regulatory circuit of EPAS1 expression in renal clear cell carcinoma
Source: Nat Commun. 2026 Feb 19;17:1764. doi: 10.1038/s41467-026-68576-0 (PMC12921326; doi:10.1038/s41467-026-68576-0)
Supplement: Supplementary file 1 — Supplementary Information [file 41467_2026_68576_MOESM1_ESM.pdf]

Supplementary Information

HIF sustain a transcriptional regulatory circuit of *EPAS1* expression in renal clear cell carcinoma

Supplementary Figure 1

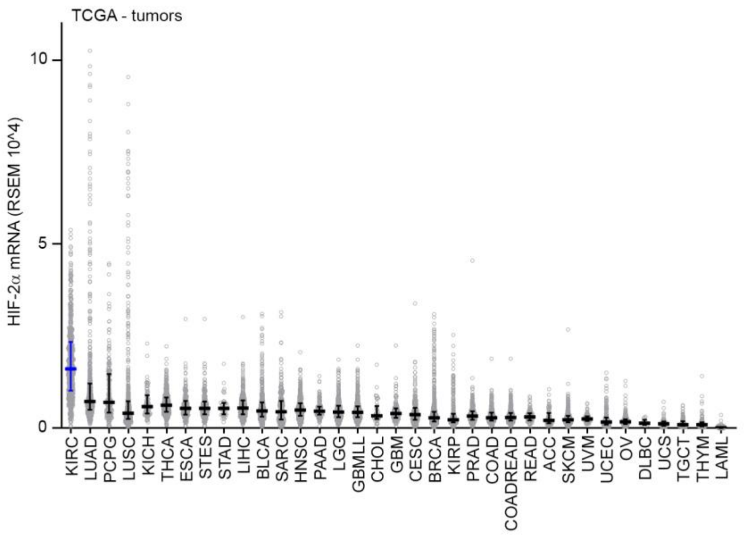

Supplementary Figure 1: HIF-2α mRNA levels are highest in TCGA KIRC samples. RSEM-normalized RNA-seq expression values for HIF-2α in different TCGA tumor entities. Kidney renal clear cell carcinoma (KIRC) is highlighted in blue. Median and interquartile range are shown. Data was generated by TCGA[1].

Supplementary Figure 2

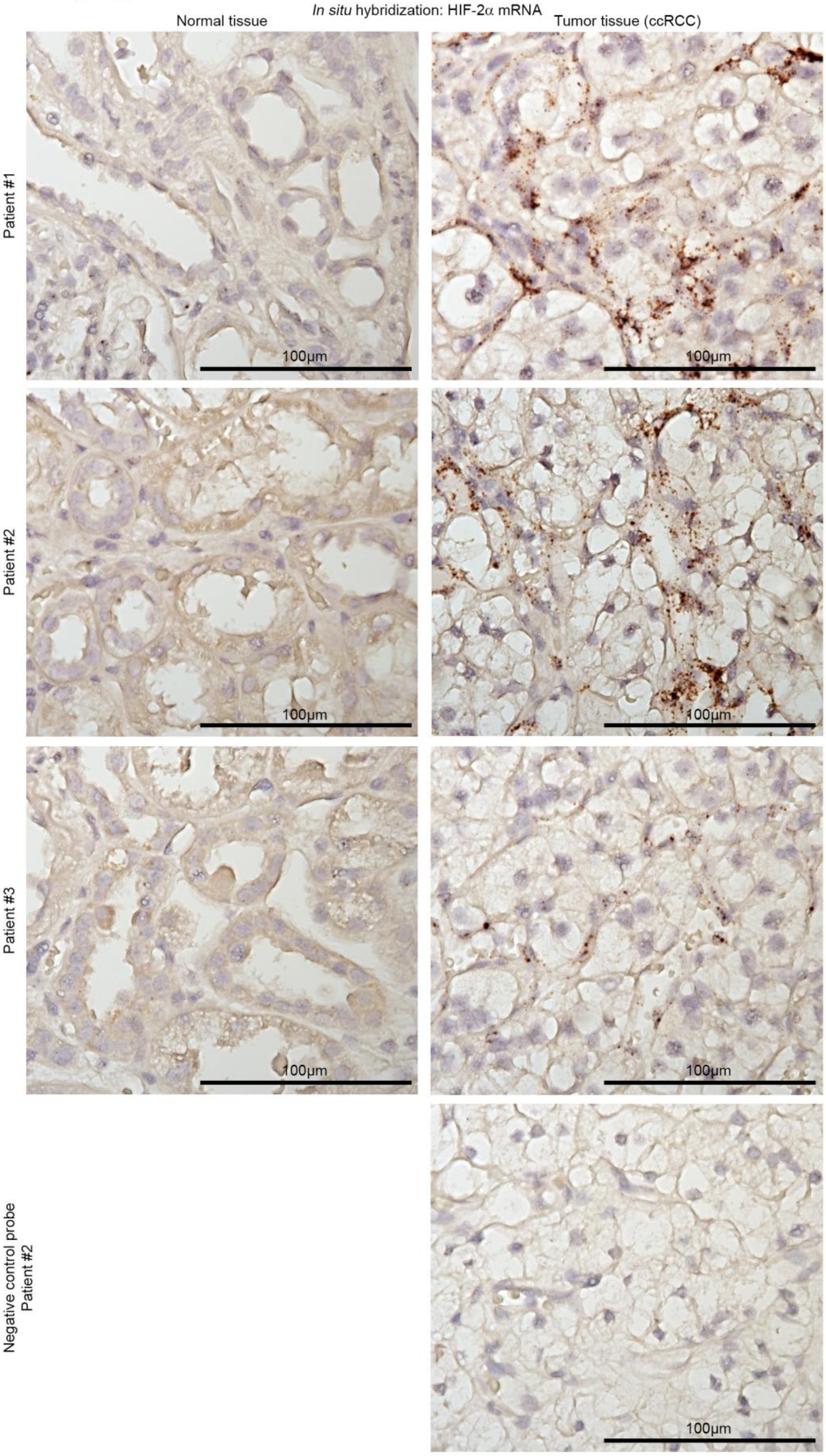

Supplementary Figure 2: HIF-2α mRNA is overexpressed in ccRCC compared to normal kidney. RNAscope® experiments using a HIF-2α specific probe in normal kidney or clear cell renal cell carcinoma (ccRCC) tissue. *In situ* hybridization experiments were repeated in samples from 3 different individuals (sample from patient #2 is the same tumor as shown in Fig. 1c, but different position on slide). A negative control probe was used as control. Scale bar, 100 µm.

Supplementary Figure 3

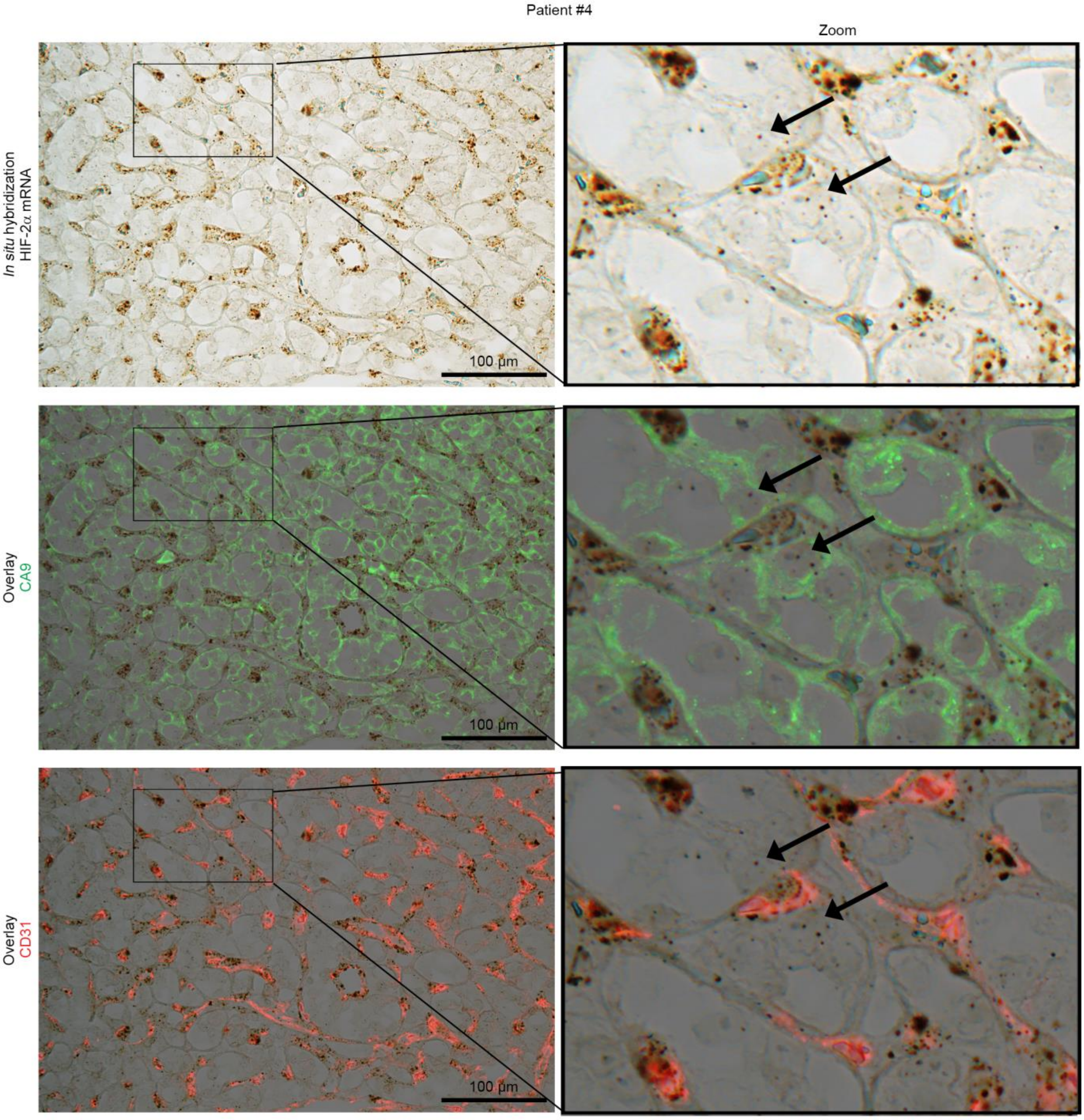

Supplementary Figure 3: HIF-2α mRNA expression is detectable in endothelial and ccRCC cells. RNAscope® experiment for HIF-2α mRNA with co-staining for markers of clear cell renal cell carcinoma (ccRCC) tumor cells (CA9, green) or endothelial cells (CD31, red) in an additional patient (patient #4). Arrows indicate HIF-2α transcripts in tumor cells with positive CA9 membrane staining. Scale bar, 100 µm.

Supplementary Figure 4

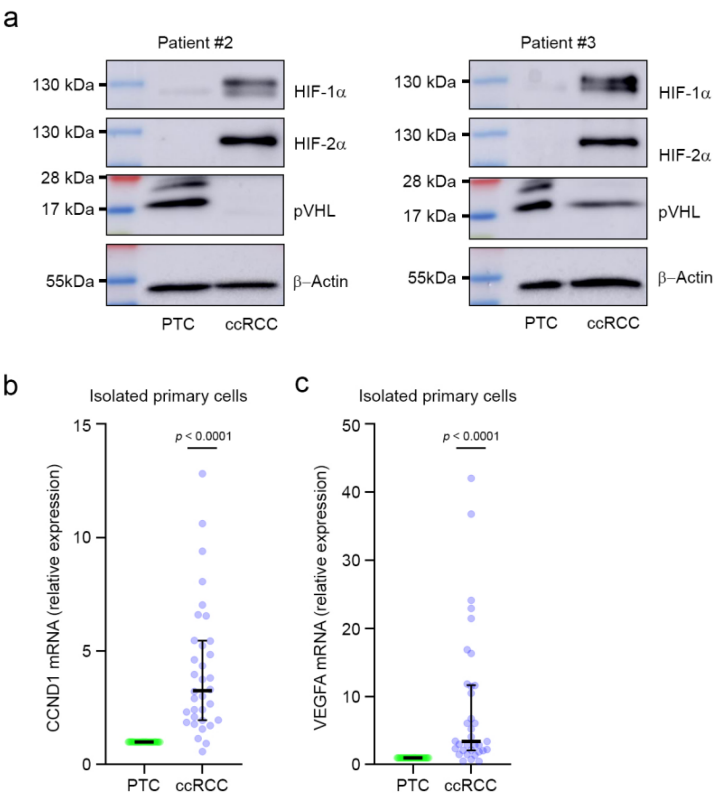

Supplementary Figure 4: HIF protein stabilization and induction of HIF target gene expression in primary ccRCC cells. a) Immunoblot analyses for HIF-1α, HIF-2α, pVHL, and β-actin protein in lysates from primary tubule cells (PTC) or tumor cells (ccRCC) isolated from tissue specimens of patient #2 and patient #3. Representative blots from 19 independent experiments with similar results. **b)** and **c)** qPCR expression analyses for CCND1 or VEGFA mRNA in lysates from primary tubule cells (PTC) or primary tumor cells (ccRCC) isolated from 34 different individuals (same patients as in Fig. 1g). Graphs show relative expression and median with interquartile range. Significance was determined by a two-tailed unpaired t-test.

Supplementary Figure 5

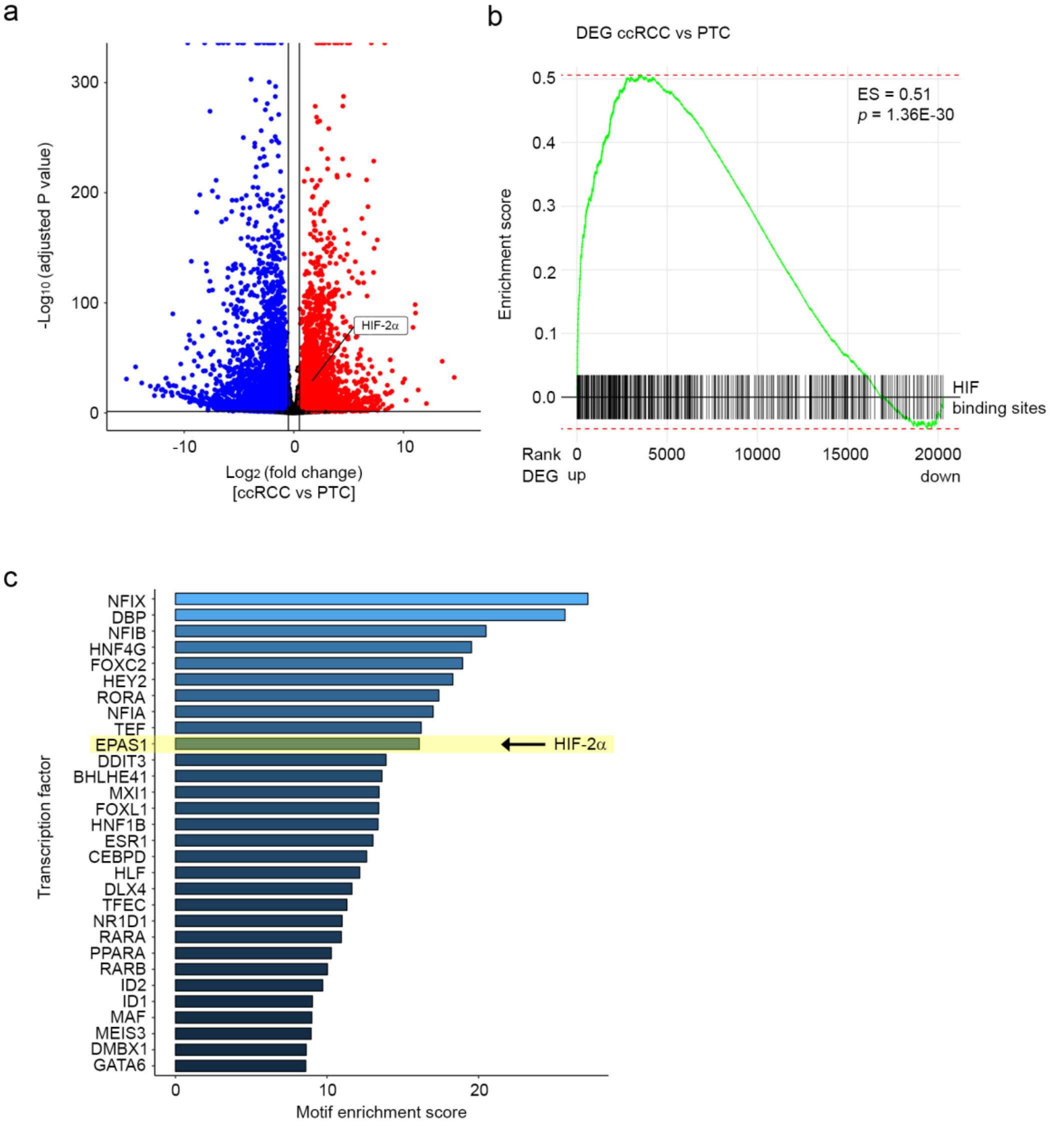

Supplementary Figure 5: Transcriptomic and epigenetic HIF-signature in primary ccRCC cells. a) Volcano plot depicting genes differentially expressed (DEG) between primary tubule cells (PTC) and primary clear cell renal cell carcinoma cells (ccRCC). RNA-seq was performed in lysates from cells from 3 different patients (Supplementary Fig. 4a and Fig. 1f). Each RNA sample was sequenced in technical duplicates. The y-axis indicates $-\log_{10}$ adjusted p-value as calculated by the Benjamini-Hochberg approach in DESeq2. **b)** Gene set enrichment analysis for Meta-HIF ChIP-seq data[2-4] and RNA-seq expression data from a). RNA-seq data were acquired from PTC and ccRCC cells of 3 different patients (n=2 technical duplicates per RNA sample). ES = enrichment score. P-value was estimated using an adaptive multilevel Monte Carlo scheme. **c)** Analysis of transcription factor binding motifs using HOMER2 in ccRCC-sites with increased accessibility ($\log_2$ fold change > 0.5, adjusted p-value < 0.05) compared to PTC as determined by ATAC-seq data and positive chromatin activity as defined by H3K27ac ChIP-seq in ccRCC. ATAC-seq was performed in PTC and ccRCC cells of 3 different patients (n=2 technical duplicates per sample). H3K27ac ChIP-seq data are from ccRCC cells of 3 different patients. Motif enrichment score was calculated by integrating motif discovery and differential expression of respective transcription factors comparing ccRCC cells and PTC.

Supplementary Figure 6

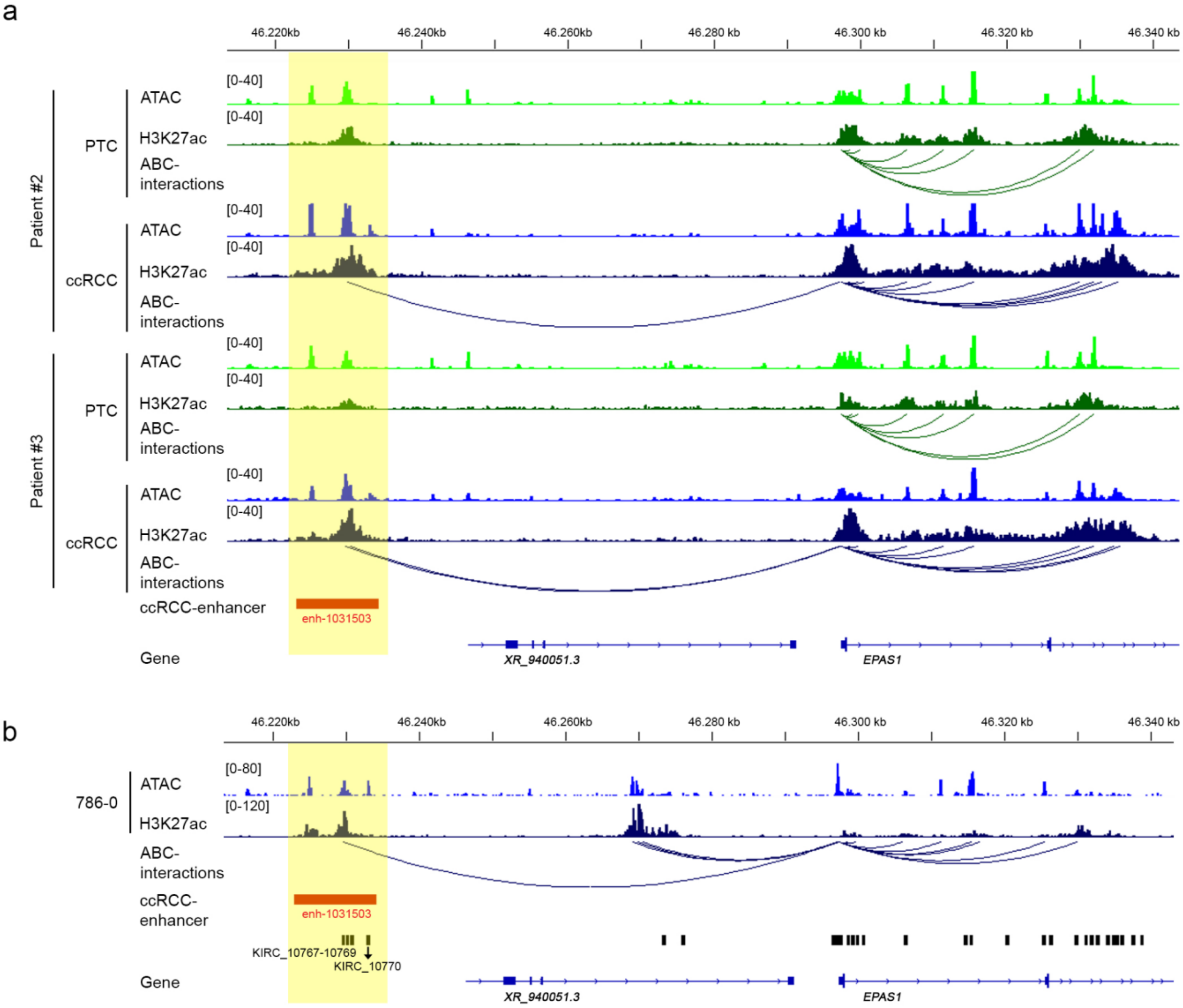

Supplementary Figure 6: ccRCC cells feature a tumor-activated *EPAS1* enhancer. a) Sequencing tracks from ATAC-seq and H3K27ac ChIP-seq experiments performed on chromatin from primary tubule (PTC) and tumor (ccRCC) cells from patients #2 and #3 at the *EPAS1* locus. The activity-by-contact (ABC) model was used to predict enhancer-promoter interactions. The ccRCC-enhancer was defined by Yao et al.[3]. ATAC KIRC track shows KIRC-specific open regions[5]. **b)** Same analysis as in a) performed with published data from the 786-0 ccRCC cell line[3,6,7].

Supplementary Figure 7

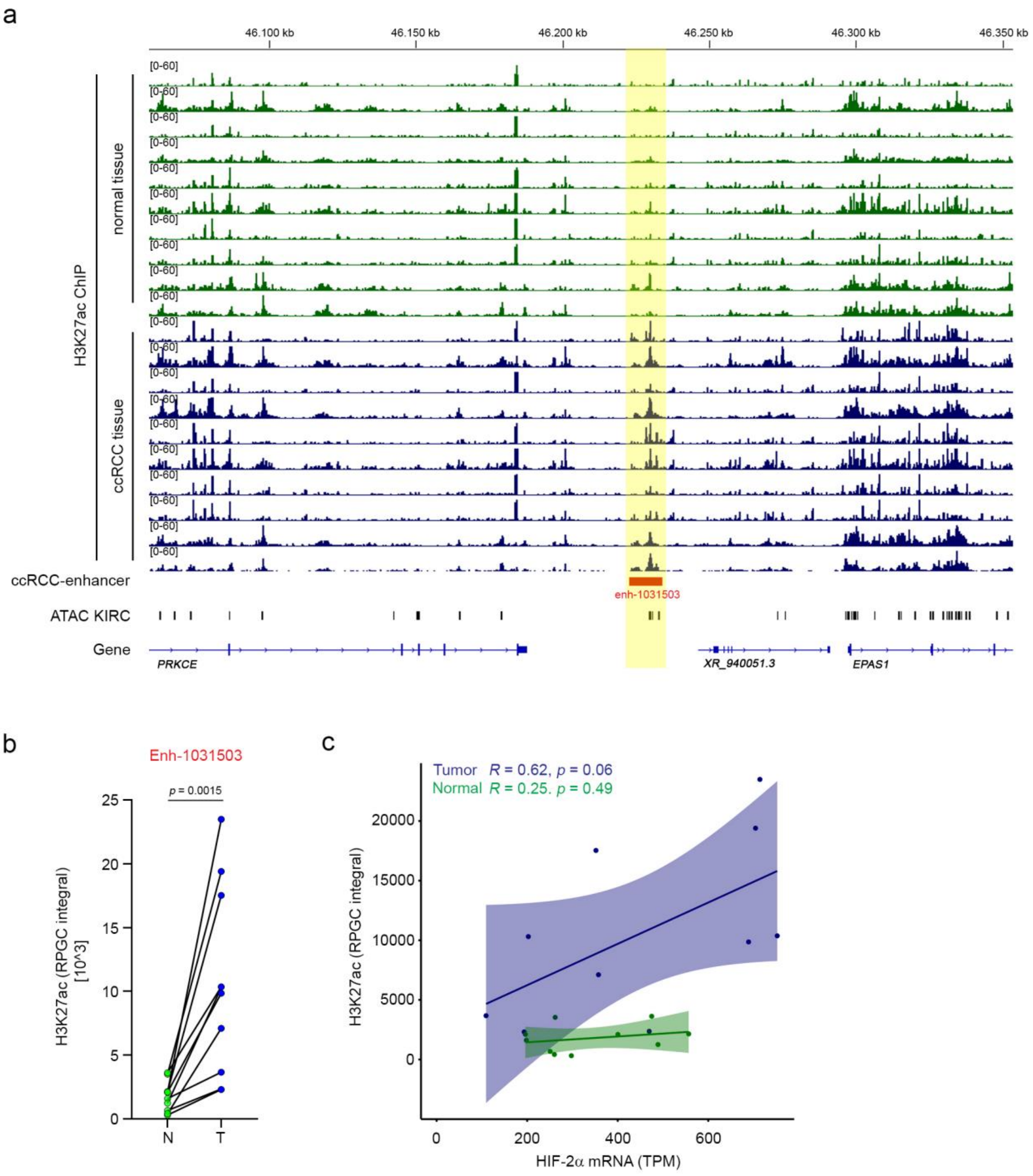

Supplementary Figure 7: Increased chromatin activity at the ccRCC-activated *EPAS1* enhancer. a) Sequencing tracks from H3K27ac ChIP-seq experiments from Yao et al.[3] performed in ccRCC tumor or adjacent normal kidney tissue from ten individuals at the *PRKCE-EPAS1* locus. The ccRCC-enhancer (highlighted in yellow) depicts a region with increased H3K27ac signal in tumor tissue (blue) in comparison to normal kidney tissue (green). ATAC KIRC track shows KIRC-specific open regions[5]. **b)** Quantification of the H3K27ac signal at the ccRCC-enhancer (enh-1031503) for normal (N) or tumor (T) tissue in samples shown in a). Tissue samples were derived from 10 different individuals[3]. Signals were normalized to reads per genomic content (RPGC) and the integral for the respective locus was calculated. Significance was assessed by a two-tailed unpaired t-test. **c)** Spearman's rank correlation analysis of HIF-2α mRNA expression (TPM: transcripts per million) and H3K27ac signals at the ccRCC-enhancer as determined in b) for normal kidney and tumor tissue. Data are from 10 different individuals[3]. Blue (ccRCC tumor tissue) and green (normal tissue) regions depict 95% confidence interval for the respective condition. Significance was assessed with a two-sided t-approximation test.

Supplementary Figure 8

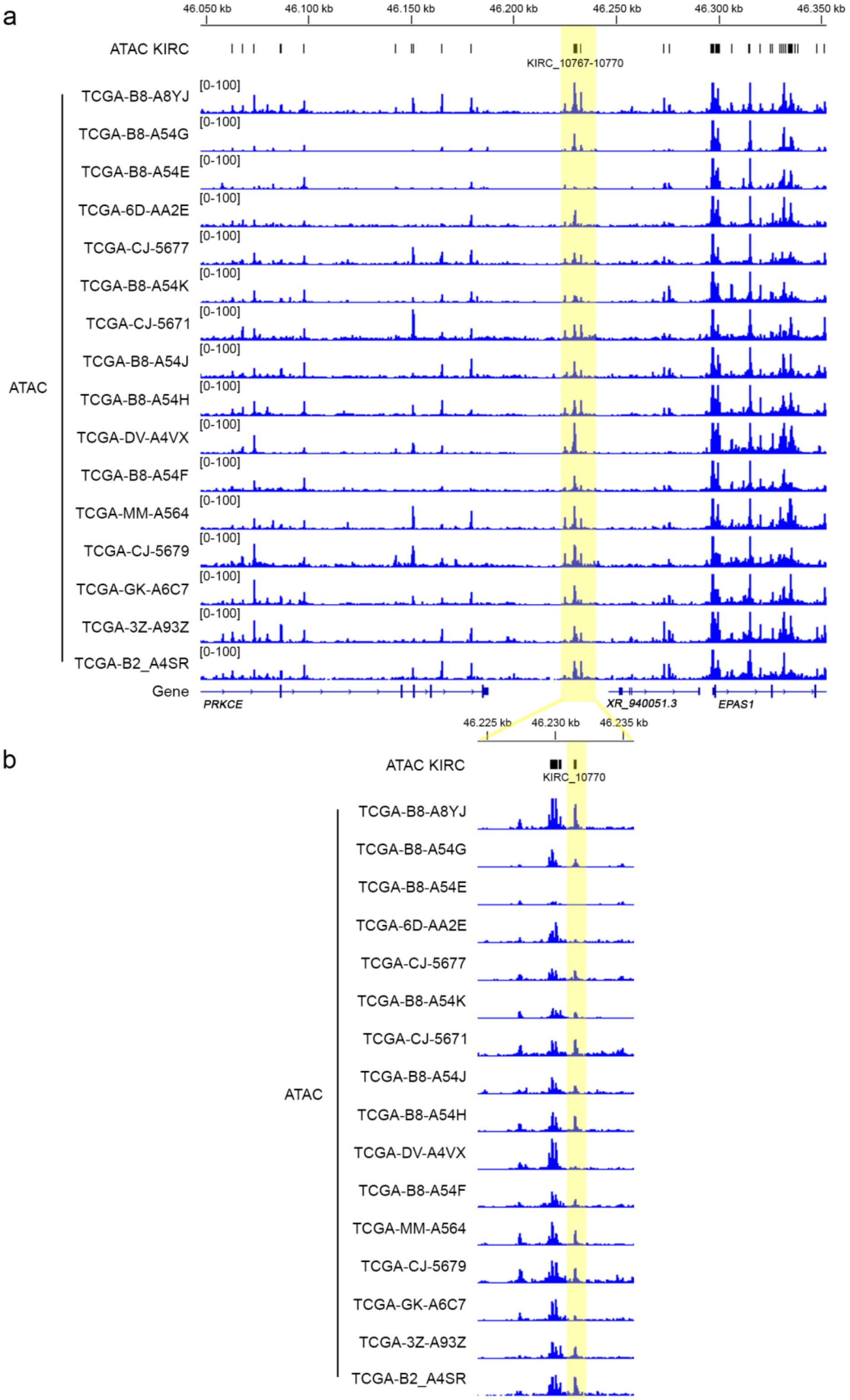

Supplementary Figure 8: Accessibility at the ccRCC-activated *EPAS1* enhancer in individual KIRC tumors. a) ATAC-seq tracks for individual KIRC samples from the TCGA dataset at the *PRKCE-EPAS1* locus[5]. **b)** Zoom of KIRC-specific open region KIRC_10770.

Supplementary Figure 9

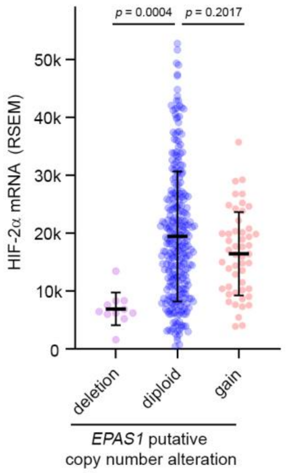

Supplementary Figure 9: HIF-2α mRNA levels and copy number gains of the *EPAS1* gene in the KIRC data set. RSEM-normalized RNA-seq expression values for HIF-2α in the TCGA KIRC cohort stratified for *EPAS1* copy number alterations (deletion: n = 11, diploid: n = 291, gain: n = 50). Data are shown as mean +/- SD. Significance was determined by one-way ANOVA followed by Bonferroni's post hoc test. Copy number alterations and corresponding RNA-seq data from the TCGA PanCancer Atlas were downloaded via the cBioPortal (https://www.cbioportal.org/, original data: https://gdc.cancer.gov/about-data/publications/pancanatlas, access 20.07.2023).

Supplementary Figure 10

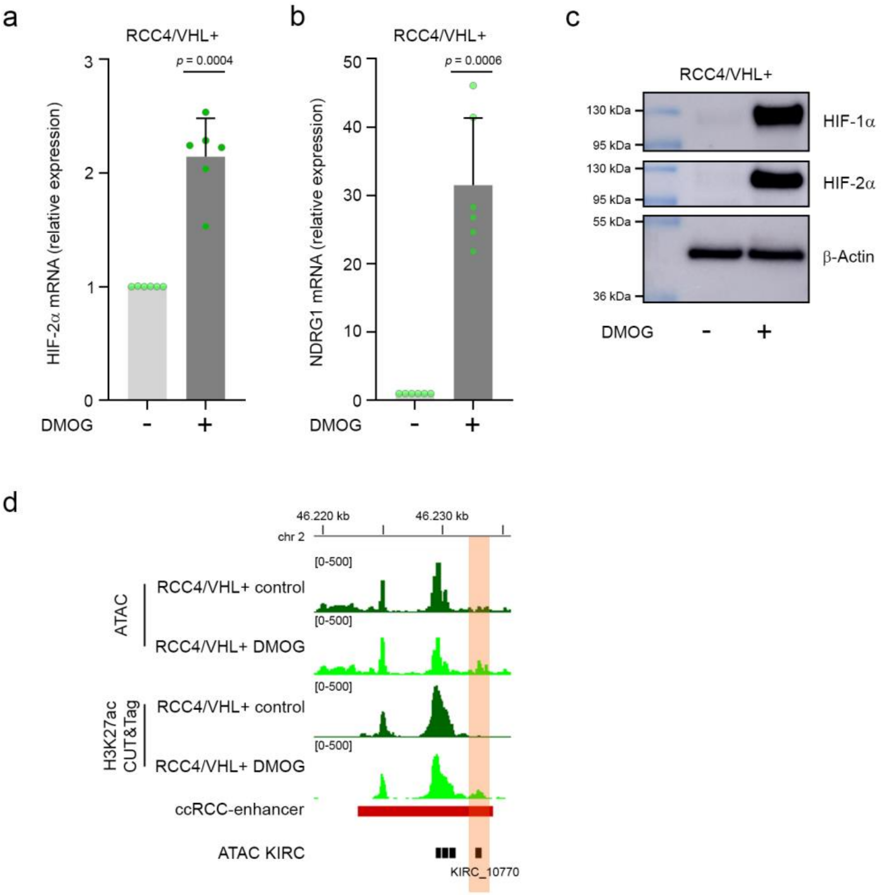

Supplementary Figure 10: Induction of HIF-2α mRNA by pharmacological HIF-stabilization in RCC4 cells re-expressing pVHL. a) and **b)** Expression qPCR analyses for HIF-2α mRNA (a) or NDRG1 mRNA (b) in lysates from RCC4 cells constitutively re-expressing pVHL (RCC4/VHL+) exposed to 1mM DMOG or control conditions for 16h. Mean + SD. Two-tailed one sample t-test with a hypothetical value of 1. n = 6 independent experiments. **c)** Immunoblot analyses for HIF-1α, HIF-2α, and β-actin in RCC4/VHL+ cells treated with 1mM DMOG or control conditions for 16h. Representative blot from two independent experiments with similar results. **d)** ATAC-seq and H3K27ac CUT&Tag-seq tracks from chromatin of RCC4/VHL+ cells exposed to 1mM DMOG or control conditions for 16h at the ccRCC-enhancer[3]. The ATAC-element KIRC_10770[5] is highlighted in orange.

Supplementary Figure 11

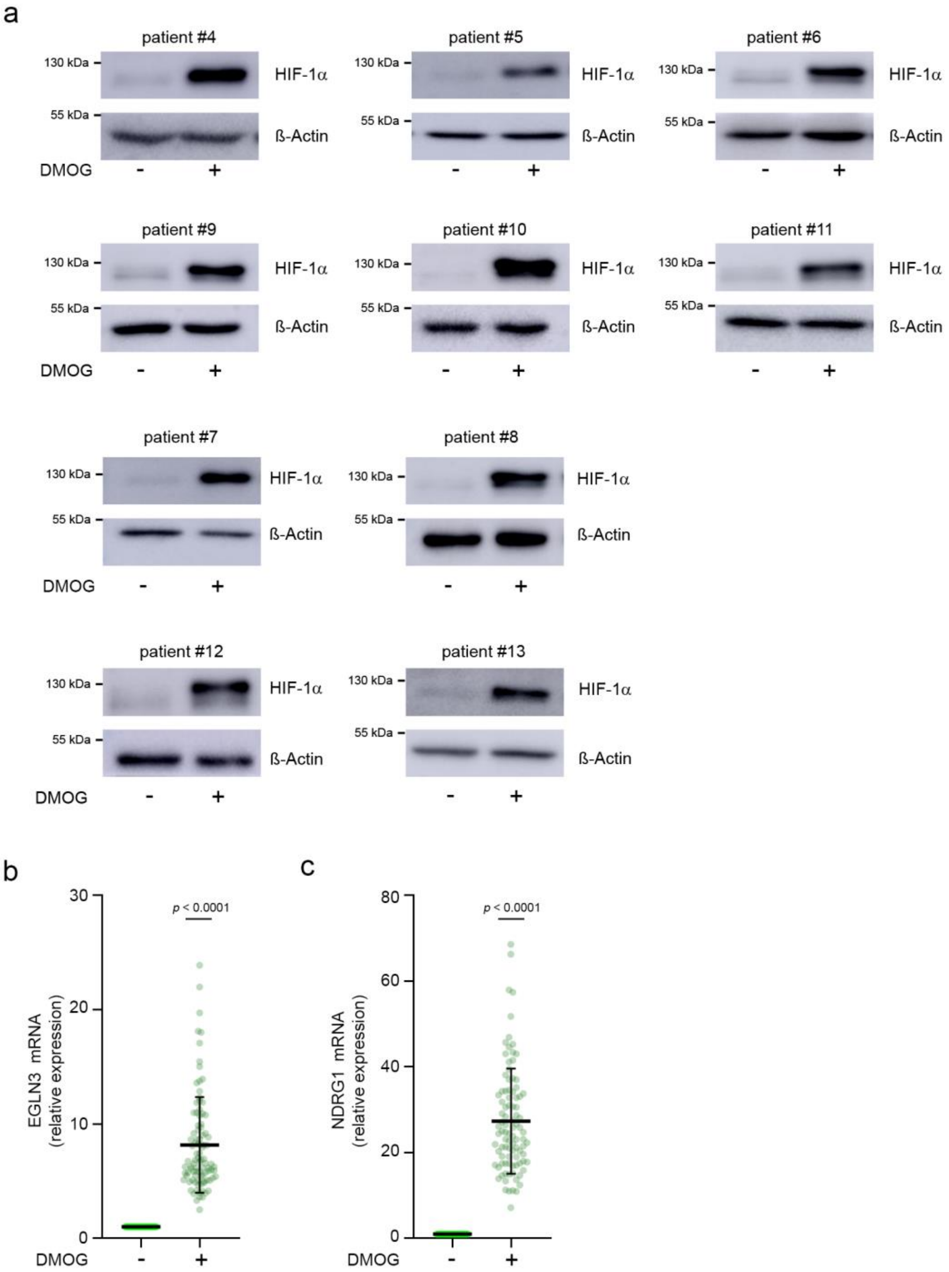

Supplementary Figure 11: HIF-stabilization in primary tubule cells. a) Immunoblot analyses for HIF-1α and β-actin in lysates from tubule cells isolated from tissue specimens. Shown is a subset of samples (10 patients out of the 105 samples used to generate qPCR expression data in Fig. 4a). Cells were treated with 1mM DMOG or control conditions for 16h. Representative blots from 40 independent experiments with similar results. **b)** and **c)** Expression qPCR analyses for EGLN3 and NDRG1 mRNA in lysates from primary tubule cells (PTC) exposed to 1mM DMOG or control conditions for 16h. Mean +/- SD. Two-tailed one sample t-test with a hypothetical value of 1. A subset of 95 PTC out of 105 PTC shown in Fig. 4a was analyzed.

Supplementary Figure 12

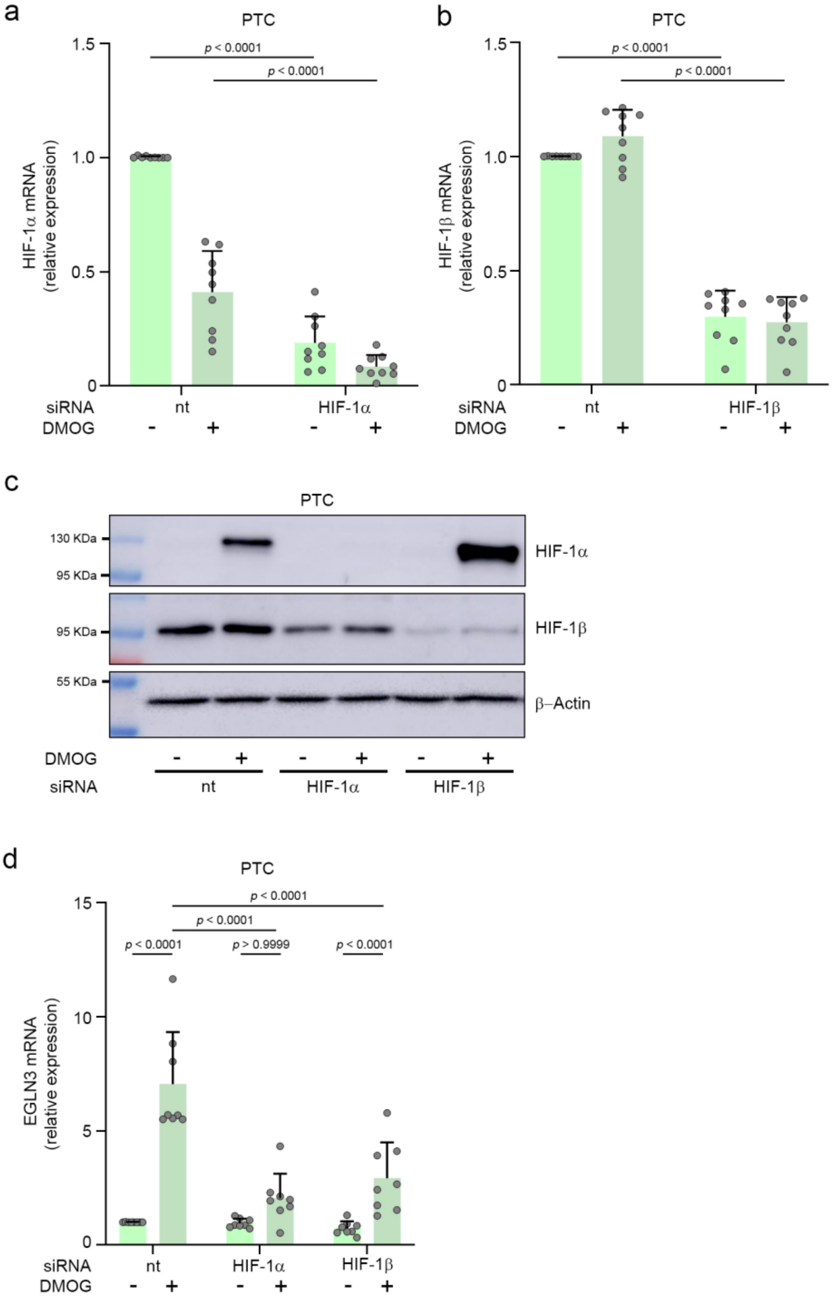

Supplementary Figure 12: Knockdown of HIF-1α or HIF-1β in primary tubule cells. a) and **b)** Expression qPCR for HIF-1α and HIF-1β mRNA in lysates from primary tubule cells (PTC) depleted for HIF-1α or HIF-1β, respectively, using siRNA and stimulated with or without 1mM DMOG for 16h. Non-targeting (nt) control siRNA was used as control. Mean + SD. Two-way ANOVA followed by Bonferroni's post hoc test. Lysates were from cells of 9 independent individuals. Same PTC samples as in Fig. 4b. **c)** Immunoblot analysis for HIF-1α, HIF-1β, and β-actin in PTC depleted for HIF-1α or HIF-1β using siRNA or transfected with non-targeting (nt) control siRNA and stimulated with or without 1mM DMOG for 16h. Representative blot from three independent experiments with similar results. **d)** Expression qPCR for EGLN3 mRNA in lysates from primary tubule cells (PTC) depleted for HIF-1α or HIF-1β, respectively. Cells were stimulated with or without 1mM DMOG for 16h. Non-targeting (nt) control siRNA was used as control. Mean + SD. Two-way ANOVA followed by Bonferroni's post hoc test. Lysates were from cells of 8 independent individuals.

Supplementary Figure 13

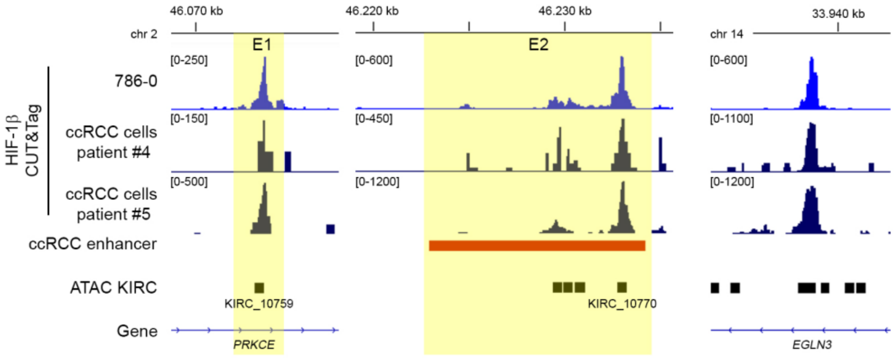

Supplementary Figure 13: HIF interacts with *EPAS1*-enhancers E1 and E2 in primary ccRCC cells. HIF-1β CUT&Tag tracks in 786-0 and primary ccRCC cells isolated from tumor nephrectomies of two independent patients (patients #4 and #5). HIF-binding sites of *EPAS1*-enhancers E1 and E2 are highlighted in yellow. Enhancer E2 is included in a region defined as a ccRCC-activated enhancer by Yao et al.[3]. ATAC KIRC track depicts KIRC-specific open regions[5]. The *EGLN3* locus serves as positive control for HIF-binding.

Supplementary Figure 14

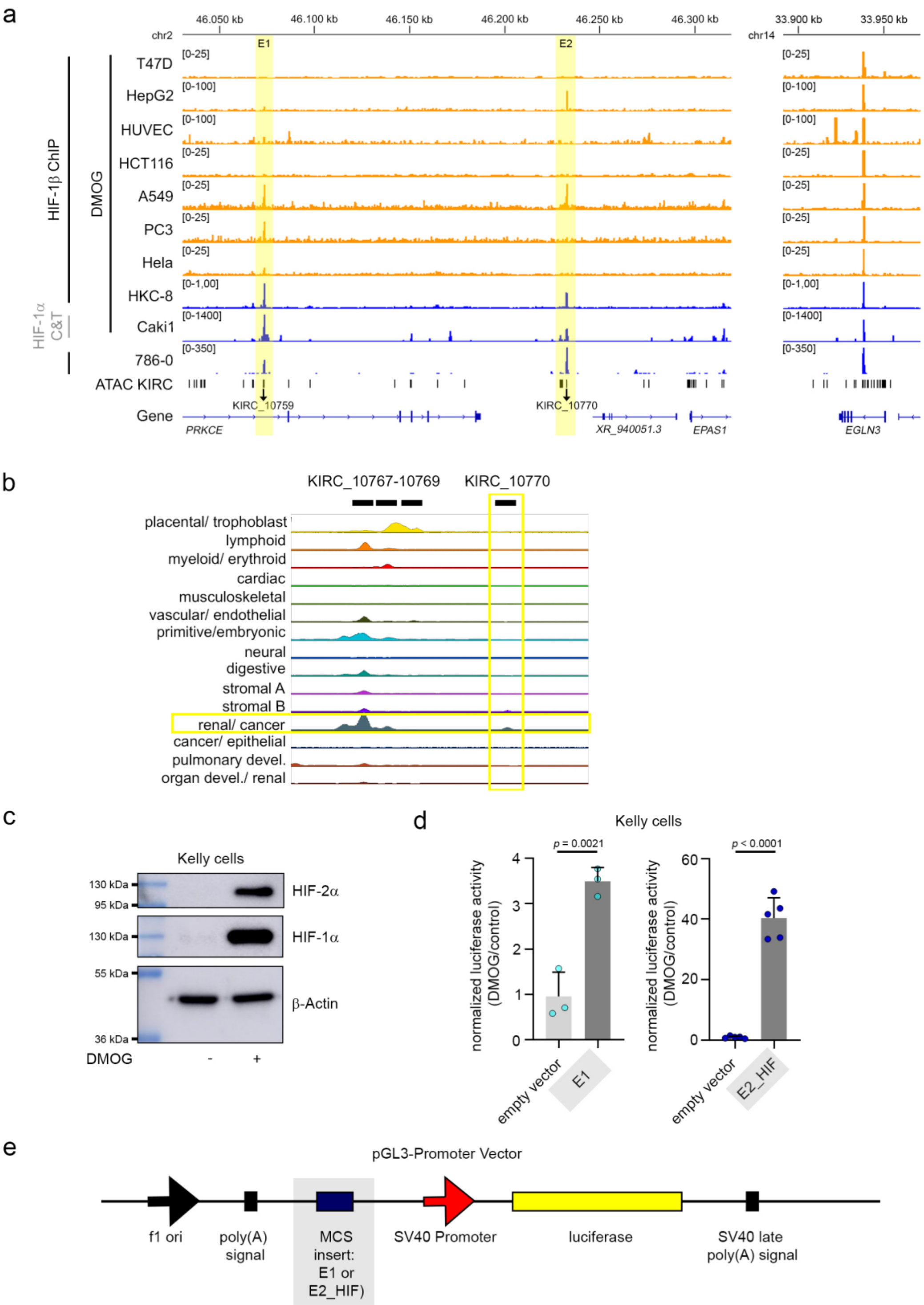

Supplementary Figure 14: HIF-interaction with ccRCC-activated *EPAS1* enhancer 2 is cell-type dependent. a) HIF-1β ChIP-seq in different cell lines and HIF-1α CUT&Tag-seq in Caki-1 cells at the *PRKCE-EPAS1* and the *EGLN3* control locus[4,8,9]. Cells were exposed to control conditions or 1 mM DMOG for 4h as indicated. Non-renal cell lines are colored in orange. Renal tubule-derived cell lines are in blue. *EPAS1*-enhancer E1 and E2 are highlighted in yellow. **b)** KIRC-specific ATAC-site KIRC_10770[5] overlaps with an accessible chromatin region defined by DNAse I hypersensitivity in the compartment "renal/cancer" from the 733 biosamples set[10]. **c)** Immunoblot analyses for HIF-2α, HIF-1α, and β-actin in lysates from Kelly cells exposed to control conditions or 1 mM DMOG for 16h. Representative blot from two independent experiments with similar results. **d)** Reporter assay using sequences covering the HIF-binding sites at *EPAS1*-enhancers E1 and E2 (E2_HIF) at the *PRKCE-EPAS1* locus. Kelly cells were transfected with pGL3-promoter vector alone or with the vector containing sequences for E1 (668bp) or E2_HIF (727 bp). Cells were exposed to 1 mM DMOG or control conditions and reporter activity was measured after 16h of stimulation. Values of luciferase activity were first normalized to activity of co-transfected β-galactosidase and subsequently to the activity of the respective control condition without HIF-stabilization. Mean + SD. Unpaired, two-tailed t test was performed. n = 3 (E1) or 4 (E2_HIF) independent transfections. **e)** Schematic of pGL3-promoter vector used for reporter assays. Enhancer sequences were cloned into the multiple cloning site (MCS).

Supplementary Figure 15

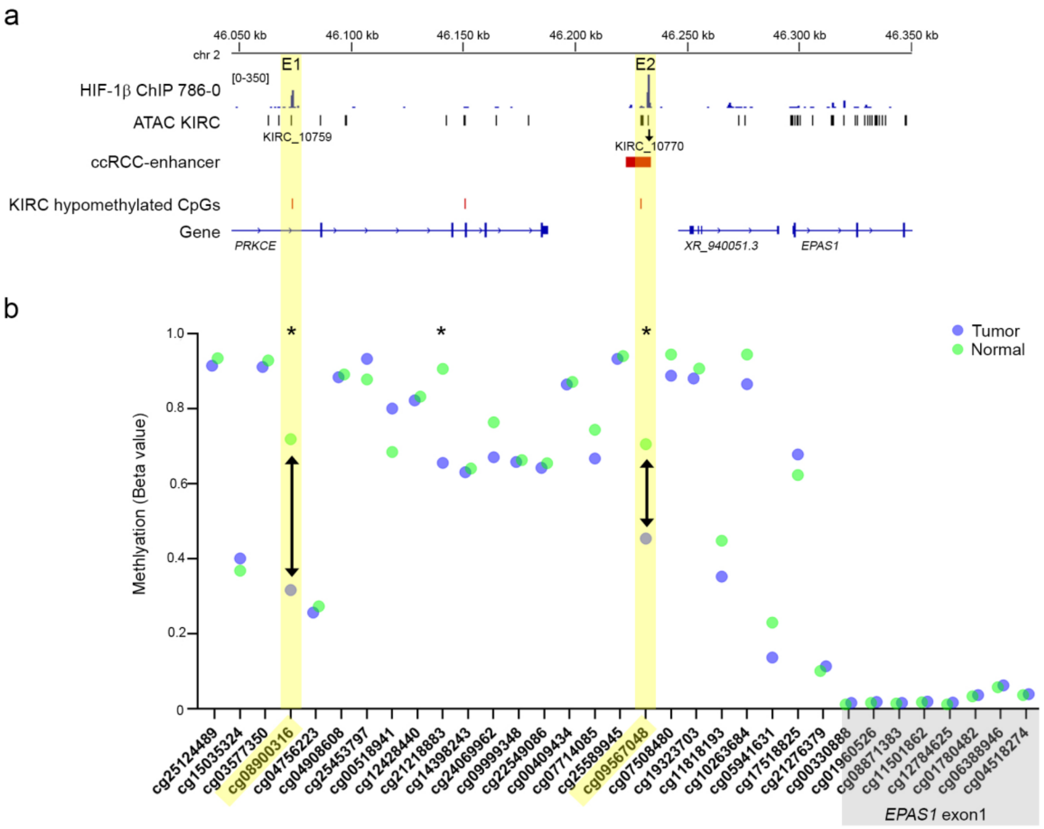

Supplementary Figure 15: *EPAS1*-enhancers E1 and E2 are located in genomic vicinity of ccRCC-hypomethylated CpGs. a) HIF-1β ChIP-seq track in 786-0 cells[2] and KIRC-specific ATAC elements[5] at the *PRKCE-EPAS1* locus. The ccRCC-enhancer as defined by Yao et al. is marked in red[3]. **b)** TCGA data for the methylation status of CpGs at the *PRKCE-EPAS1* locus in the KIRC dataset (derived from 160 normal and 324 tumor tissues) were downloaded from http://maplab.imppc.org/wanderer/ (access 12.04.2024). Significantly hypomethylated CpGs in the tumors (Beta-value cutoff 0.25, adjusted p-value cutoff 0.01, marked with *) were accessed *via* http://www.bioinfo-zs.com/smartapp/ (28.11.23). Arrows indicate differences in the methylation status of CpGs in genomic vicinity of the *EPAS1*-enhancers E1 and E2. CpG probes located within the first exon of the *EPAS1* gene are marked in grey.

Supplementary Figure 16

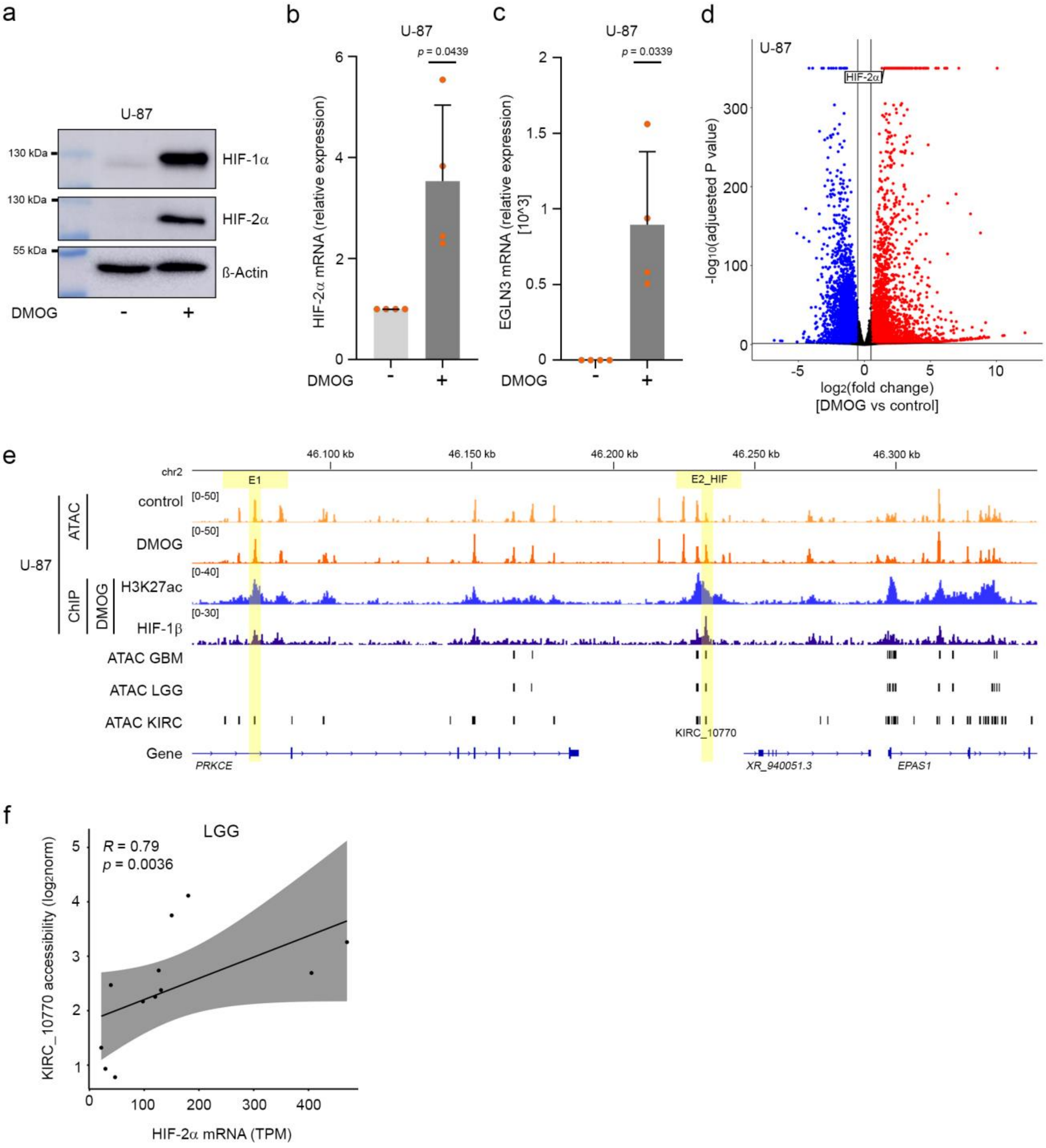

Supplementary Figure 16: HIF-binding to *EPAS1*-enhancer E2 in glioblastoma cells. a) Immunoblot analyses for HIF-1α, HIF-2α, and β-actin in lysates from U-87 malignant glioma cells exposed to control conditions or 1 mM DMOG for 16h. Representative blot from two independent experiments with similar results. **b)** and **c)** qPCR expression analyses for HIF-2α (b) or EGLN3 (c) mRNA, respectively, in U-87 cells exposed to control conditions or 1 mM DMOG for 16h. Mean + SD. Significance determined by a two-tailed one sample t test with a hypothetical value of 1. n = 4 independent experiments. **d)** Volcano plot showing differentially expressed genes in RNA-seq experiments using U-87 cells exposed to 1 mM DMOG for 16h or control conditions. HIF-2α transcript is indicated. One RNA sample was generated per condition and sequenced in technical duplicates. Y-axis indicates $-\log_{10}$ adjusted p-value as calculated by the Benjamini-Hochberg approach in DESeq2. **e)** Sequencing tracks from ATAC-seq, H3K27ac and HIF-1β ChIP-seq experiments performed in U-87 cells cultured under control conditions or exposed to 1 mM DMOG as indicated. ATAC GBM, ATAC LGG and ATAC KIRC tracks depict glioblastoma-, glioma- and clear cell renal cell carcinoma-specific accessible elements as determined in the TCGA dataset[5]. KIRC_10770 coincides with GBM- and LGG-accessible ATAC-sites. **f)** Spearman's rank correlation analysis of HIF-2α mRNA expression (TPM: transcripts per million) and accessibility at KIRC_10770 in the LGG data set[1,5]. Values are from samples for which both analyses were available (n = 12). Gray region depicts 95% confidence interval. Significance was assessed with a two-sided t-approximation test.

Supplementary Figure 17

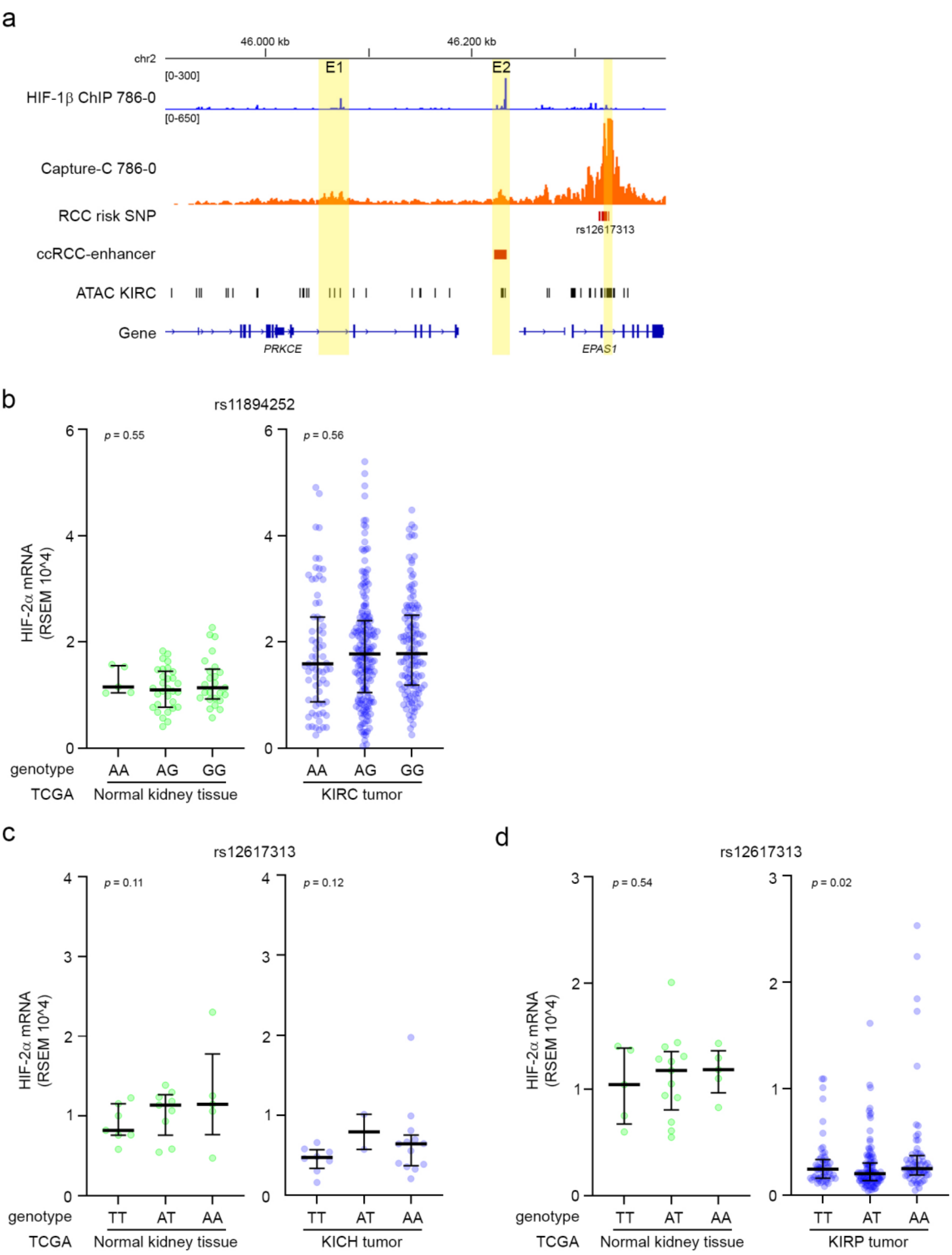

Supplementary Figure 17: Genetic predisposition and HIF-2α expression. a) HIF-1β ChIP-seq[2] and Capture-C track[8] of 786-0 cells at the *PRKCE-EPAS1* locus. RCC-associated SNP rs12617313 as defined by Han et al.[11] and SNPs in high LD ($R^2 > 0.8$) within the intronic region of *EPAS1* are indicated. Capture-C tracks reveals chromatin interactions between a regulatory element close to the SNP (anchor site) and *EPAS1*-enhancers E1 and E2 (highlighted in yellow). The ccRCC-enhancer was described by Yao et al.[3]. ATAC KIRC track shows KIRC-specific accessible regions[5]. **b)** Genotype-expression analysis for GWAS RCC risk SNP rs11894252 and HIF-2α mRNA for normal and tumor tissue determined in the TCGA KIRC cohort[1]. Normal tissue: Genotype AA n=5, AG n=30, GG n=25. Tumor tissue (KIRC): Genotype AA n=71, AG n=184, GG n=132. Data shown are median and interquartile range. χ^2-test; p-value as indicated. **c)** Genotype-expression correlation for rs12617313 and HIF-2α mRNA for normal and tumor tissue determined in the TCGA cohort for chromophobe kidney cancer (KICH, Normal tissue: Genotype TT n=7, AT n=9, AA n=5. Tumor tissue: Genotype: TT n=8, AT n=2, AA n= 13)[1]. **d)** Genotype-expression correlation for rs12617313 and HIF-2α mRNA for normal and tumor tissue determined in the TCGA cohort for papillary kidney cancer (KIRP, Normal tissue: Genotype TT n=5, AT n=13, AA n=5. Tumor tissue: Genotype TT n=54, AT n=106, AA n=65)[1]. *χ^2-test, p-value as indicated. Data shown are median and interquartile range.

Supplementary Figure 18

a

DOR with reduced accessibility: 786-0 HNF-1β ko versus nt

Rank	Motif	P-value	log P-pvalue	% of Targets	% of Background	STD(Bg STD)	Best Match/Details
1	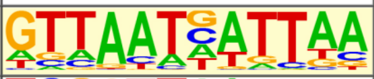	1e-845	-1.947e+03	23.29%	2.18%	49.7bp (84.6bp)	HNF1b(Homeobox)/PDAC-HNF1B-ChIP-Seq(GSE64557)/Homer(0.992)
2	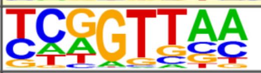	1e-83	-1.930e+02	19.85%	10.75%	57.6bp (77.9bp)	AT3G10030/MA1662.1/Jaspar(0.814)
3	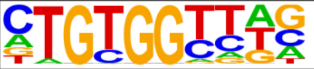	1e-61	-1.414e+02	32.68%	22.72%	48.5bp (67.2bp)	RUNX(Runt)/HPC7-Runx1-ChIP-Seq(GSE22178)/Homer(0.957)
4	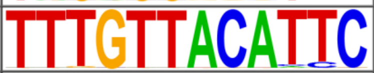	1e-52	-1.209e+02	0.45%	0.00%	61.6bp (5.0bp)	DMRTA2/MA1478.1/Jaspar(0.791)
5	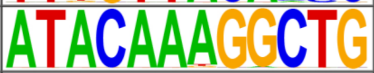	1e-49	-1.150e+02	0.43%	0.00%	57.6bp (18.2bp)	Sox3/MA0514.1/Jaspar(0.710)
6	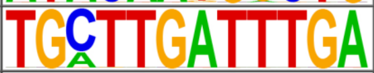	1e-47	-1.091e+02	0.41%	0.00%	62.3bp (0.0bp)	onecut/dmmpmm(Noyes_hd)/fly(0.742)
7	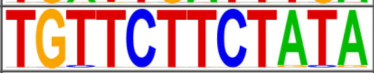	1e-44	-1.033e+02	0.39%	0.00%	35.7bp (0.0bp)	SFL1/MA0377.1/Jaspar(0.727)
8	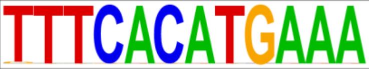	1e-34	-7.860e+01	0.36%	0.00%	66.7bp (2.4bp)	INO4/INO4_YPD/4-INO4,37-INO2(Harbison)/Yeast(0.840)
9	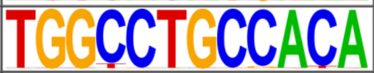	1e-33	-7.628e+01	0.49%	0.01%	45.9bp (53.4bp)	MET32/MA0334.1/Jaspar(0.653)
10	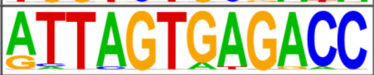	1e-30	-7.061e+01	0.43%	0.01%	47.7bp (102.6bp)	M1BP(Zf)/S2R+-M1BP-ChIP-Seq(GSE49842)/Homer(0.719)

b

DOR with reduced accessibility: 786-0 PAX8 ko versus nt

Rank	Motif	P-value	log P-pvalue	% of Targets	% of Background	STD(Bg STD)	Best Match/Details
1	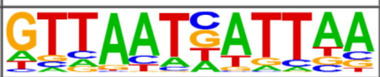	1e-578	-1.332e+03	11.16%	2.13%	50.2bp (69.0bp)	HNF1b(Homeobox)/PDAC-HNF1B-ChIP-Seq(GSE64557)/Homer(0.995)
2	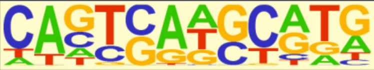	1e-432	-9.959e+02	26.61%	12.37%	48.4bp (62.8bp)	Pax8(Paired,Homeobox)/Thyroid-Pax8-ChIP-Seq(GSE26938)/Homer(0.884)
3	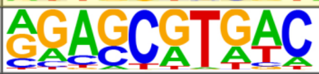	1e-121	-2.805e+02	13.28%	7.42%	52.1bp (60.3bp)	PAX5/MA0014.3/Jaspar(0.829)
4	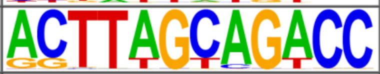	1e-81	-1.874e+02	0.36%	0.00%	56.3bp (55.3bp)	dsx-F/dmmpmm(Pollard)/fly(0.666)
5	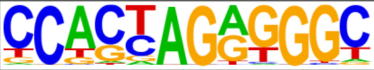	1e-70	-1.624e+02	4.72%	2.15%	39.5bp (46.0bp)	BORIS(Zf)/K562-CTCFL-ChIP-Seq(GSE32465)/Homer(0.921)
6	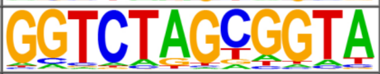	1e-70	-1.613e+02	0.44%	0.01%	50.3bp (41.3bp)	Smad4/MA1153.1/Jaspar(0.662)
7	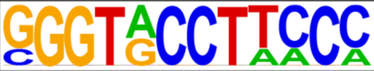	1e-69	-1.593e+02	0.34%	0.01%	60.4bp (37.5bp)	PCBP2(KH)/Homo_sapiens-RNCMPT00044-PBM/HughesRNA(0.715)
8	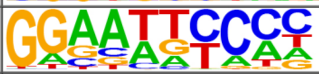	1e-61	-1.420e+02	7.03%	3.95%	50.5bp (60.4bp)	REL/MA0101.1/Jaspar(0.901)
9	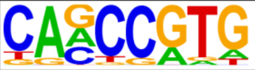	1e-55	-1.269e+02	11.82%	7.92%	51.5bp (59.6bp)	RAP1/RAP1_YPD/85-RAP1(Harbison)/Yeast(0.718)
10	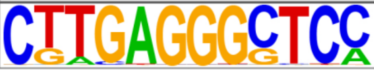	1e-48	-1.126e+02	0.38%	0.02%	47.3bp (66.5bp)	Nkx2-5(var.2)/MA0503.1/Jaspar(0.679)

Supplementary Figure 18: Enrichment of transcription factor motifs in ATAC-sites with reduced accessibility upon HNF-1β or PAX8 knock-out. a) HOMER *de novo* motif analysis of differentially open regions (DOR) with reduced accessibility as defined by ATAC-seq (log_2 fold change of < -0.5, adjusted p-value of < 0.05) comparing HNF-1β knock-out with control single clones of 786-0 cells. The HNF-1β motif is highlighted in yellow. **b)** Same analysis as in a) analysing ATAC-sites with decreased accessibility (log_2 fold change of < -0.5 with, adjusted p-value of < 0.05) comparing PAX8 knock-out with control single clones of 786-0 cells. The PAX8 motif is highlighted in yellow.

Supplementary Figure 19

a

E2

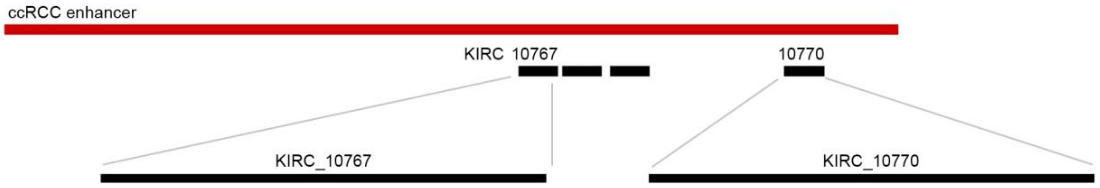

KIRC_10767

AGATTTCTTTCCTTCTCTGATTATC — E2_PAX8 sgRNA#1
CTCACCACTAGGTGGGATGGAAGC
TGTCTACCAGAGTCTGCTGCAGGT
TAACCCTTCCCCTGCCCTCATTCAA — E2_PAX8 sgRNA#2
GGACATGACAGGGATGTTCCAGGT
AAATGTGCCCCCTTGGCTTGAGAC
AGCACTTGTGGTAATGGAATAGCT
TCCTCTGCTATGGCCTGCTTGTGT
TAGTTCACTGAGAATGGACTCCCT
GTGGGTTCGTAGAGTGCAACAATT
HNF-1β motif: GAAGAGCTTGAA**TAATGGTTAAC**C — E2_HNF-1β sgRNA
AGGATGAAGTCACATCCGAATATG
CGTCATCTCACCACCTCCTTTGCT
GCAGTCCGAAAACCCCACTGAGTC
TTTGTTCTTTTTCCATCCGTCTGTC
TCTCTTGGTATCTGTCTCTCTCTTT
CTCATCACCCCTTCAGGGTATTGA
TGCTTATTGATACTGGATTTTCTTT
CTCACTCCAGTTTTTCCTGTCTCTA
TCCACCTGTCTTTCTCCCTCCAGCT
CTCCCTCACTCTCTC

KIRC_10770

TAAATGCTGGGGGCTCAAATACTG
CCCCTCCACCCCCATTTCACCCTG
CACCAGCTCCTCCTCAGTCAGGTC
TAGGGTCCTGCCTCTGTCCCAGTG
GCCACCTGGCTGTGGGGGCAGGG
TGGGCCACCCAGGAACCTGTGTG
GTCAGGGCTCTGCATGGAGGGTCT
GCCAGGGCAGCCTGGGATGTGGC
TGGGCTGCTGCTGTGGACTGGGT
HRE1: GGGGTGGGAG**GCGTG**GACAGCTC
HRE2+3: CA**GCGTG**CTCTCCCT**CACGT**CCTT — E2_HIF sgRNA#1
GAAGTTTGCCCATCCTCTGACTCA — E2_HIF sgRNA#2
GCTCCAGCATCTCAATTCGTGGAT
GAATGCCCTGGTCCTAGACAGAGA
GCCAATTCAGCCCAGCAGGGTTTC
TGGAGGGCTCCCAGGAGGGGGAG
TCCCGGAACATGGCCCGGGACAG
TGAAGTTGGGGTGACGGCCAGTTC
ACTCCAGCTGCAGCCTAGGACACA
TTCCTCTCCATTCCCACCCCCACC
TCACATTCTGCTGGAGCCCTCCAT
AGTGC

b

E1

KIRC_10759

GGAAATCCTTCAAATATTTTAAGAGTTTCTCTTCAGTTTGTCCTTTGTCTGATACTTAT
TTGCCCATTCATTCAGTTCAGACTGCCAGTCTCCTCTAACACTGGATTTACTGGGGC
TGGCTGTTGCACTTCCCTCAGCTGCTTCTCGGAGGTCCTGGCTTCTCCTCGCCCTC
CTGTTTGCCCTCAGTCAGCGTTCAGTGTAGTGCACAGTGTCTGACACCACACTGACC
GTGTGGTCTGACCAGCAGAACCCAACAGGAGCAGCAGGAGCGGTTGCTTCCTGCAT
TCCAGGCTCTGTCCTGCTTCAACTGAAGACTGTTCTGAGACTAATCAGGGACCCAAA
CTGATGCAGACAGAAATCTCCGTGGCAGATCAGGAAGGGAAAATGTCAGTGTGGTT
HRE1: GAATTTACCAGGCATGAGT**CACGT**CCTGGTAAATGAATACATCTGGGGATGGAAGA — E1 sgRNA#1
GAGACACCGTCCTTCCAGTATTCCAAGTGGAGAAATACTGTTTCACTGGATACCTGA
HRE2+3: GTAACTGCTGCACAGACC**ACGC**TATG**CACGT**ATCATCAGGTCTGCAAGCTGAACAG — E1 sgRNA#2
HRE4: ATGCTCT**CACGT**GTCATCTCTTAGAGGTAGGATCAGTCCTGGCTGCTATACTCAAGC
AGGAAAGGTAAGAGGGGGCAGCTTTTTTTCCCTCTCTTTGTGTAGTGTGAGCAACCT
AAATATTTCCATCTAAGCAGGTTATGGTTCCTTCCCACAATTGAAGGGAAATATTGAT
GTGCCTGTTTATTGGGG

Supplementary Figure 19: Transcription factor motifs and target regions of sgRNAs used for knock-out experiments at *EPAS1* enhancer E1 and E2. a) DNA sequences of the ATAC-KIRC[5] elements 10767 and 10770 at the ccRCC *EPAS1*-enhancer E2. KIRC_10767 binds PAX8 (no motif detected) in ChIP-seq experiments and HNF-1β (respective motif is marked in bold). Positions of sgRNA targeting the PAX8 and the HNF-1β binding site are underlined. KIRC_10770 carries three HREs (marked in bold). Positions of sgRNAs targeting the HIF-1β binding site are underlined. **b)** DNA sequence at the *EPAS1*-enhancer E1. The sequence covered by ATAC-KIRC element 10759 is highlighted in grey. Hypoxia-responsive elements (HRE) are marked in bold letters. Positions of sgRNAs targeting HREs in E1 are underlined.

Supplementary Figure 20

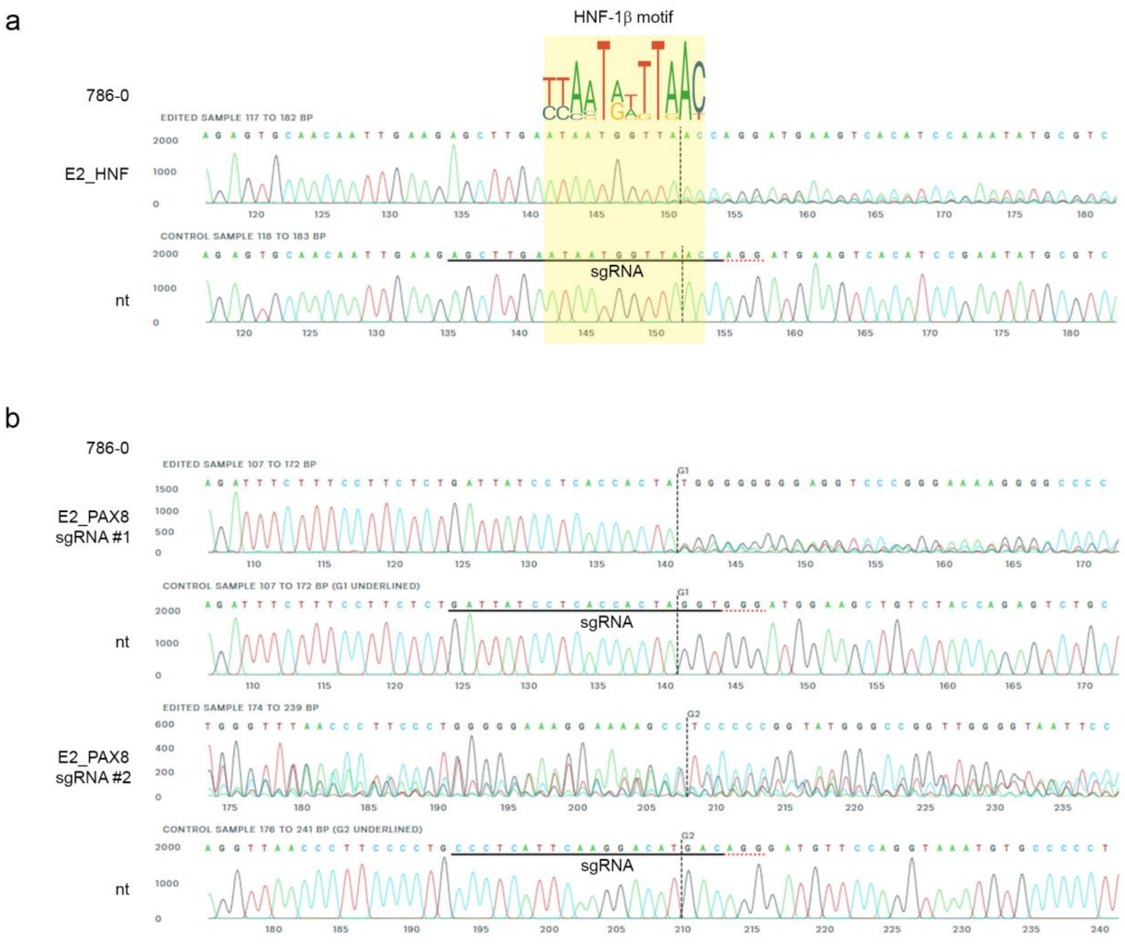

Supplementary Figure 20: "Inference of CRISPR Edits" (ICE) analysis for HNF-1β and PAX8 binding site knock-out at *EPAS1*-enhancer E2 in 786-0 cell pools. a) Sanger sequencing tracks of control and edited DNA isolated from 786-0 cell pools treated with a sgRNA targeting the HNF-1β motif at *EPAS1*-enhancer E2 (E2_HNF) or a non-targeting control sgRNAs (nt). The target region of the sgRNA and the transcription factor motif are highlighted. The HNF-1β sequence logo was downloaded from JASPAR 2022 (Matrix profile MA0153.1). **b)** Sanger sequencing tracks of control and edited DNA isolated from 786-0 cell pools treated with sgRNAs targeting the center of the PAX8-ChIP-seq peak as published by Patel et al. (E2_PAX8 sgRNA #1 and #2) or non-targeting control sgRNAs (nt).

Supplementary Figure 21

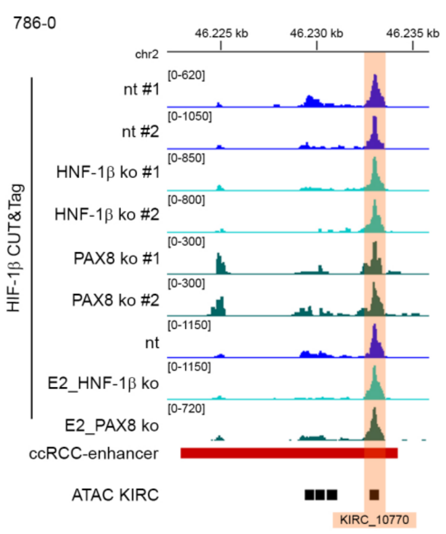

Supplementary Figure 21: Preserved HIF-1β binding at *EPAS1*-enhancer 2 upon interference with HNF-1β or PAX8 signaling. HIF-1β CUT&Tag-seq tracks at *EPAS1*-enhancer 2 in 786-0 cells subjected to HNF-1β or PAX8 knock-out (HNF-1β ko, PAX8 ko; 2 different single clones of cells per condition) or knock-out of the binding site for the respective transcription factor within *EPAS1*-enhancer 2 (E2_HNF-1β ko, E2_PAX8 ko; one cell pool per condition). Tracks from 786-0 cells generated with a non-targeting sgRNA (nt) are shown as a control. The ccRCC-enhancer as defined by Yao et al. is shown in red[3]. ATAC KIRC track shows KIRC-specific open regions[5].

Supplementary Figure 22

a

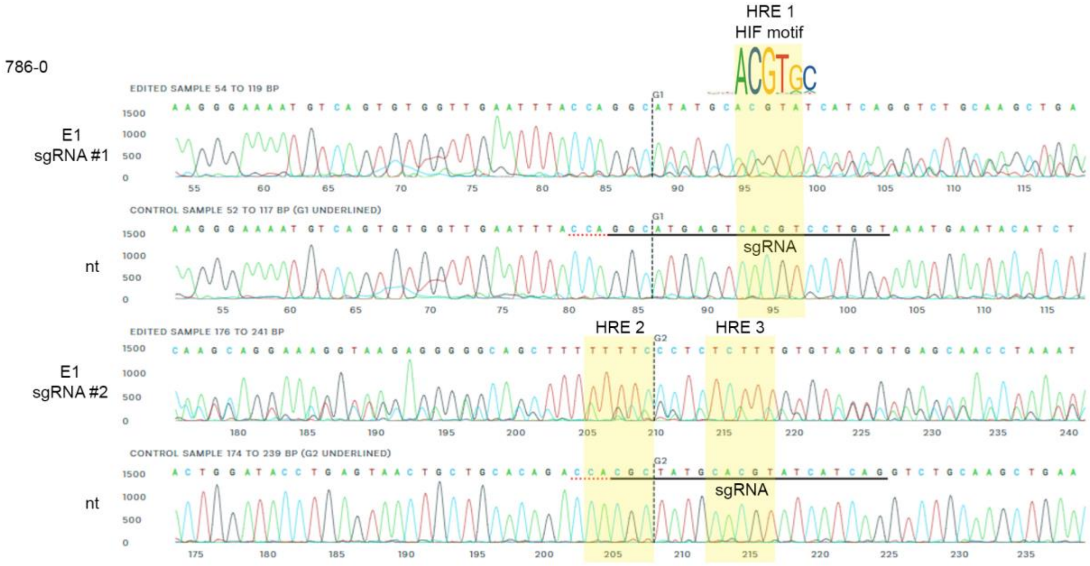

b

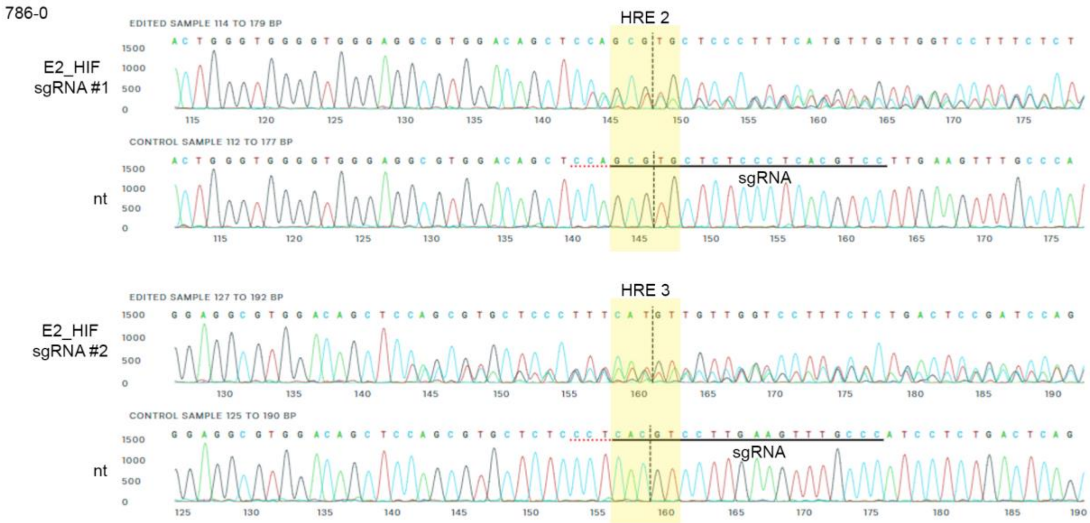

Supplementary Figure 22: "Inference of CRISPR Edits" (ICE) analysis of HIF binding sites knock-out at *EPAS1*-enhancers 1 and 2 in 786-0 cell pools. a) Sanger sequencing tracks of control and edited DNA isolated from 786-0 cell pools treated with sgRNAs targeting the hypoxia-responsive elements 1-3 (HRE 1-3) at *EPAS1*-enhancer 1 (E1 sgRNA #1 and #2) or non-targeting control sgRNAs (nt). The target region of the sgRNAs and the transcription factor motifs are highlighted. The HIF sequence logo was downloaded from JASPAR 2022 (Matrix profile MA1106.1). **b)** Sanger sequencing tracks of control and edited DNA isolated from 786-0 cell pools treated with sgRNAs targeting the hypoxia-responsive elements 2 and 3 (HRE 2 and 3) at *EPAS1*-enhancer 2 (E2_HIF sgRNA #1 and #2) or non-targeting control sgRNAs (nt). The target region of the sgRNA and the transcription factor motifs are highlighted.

Supplementary Figure 23

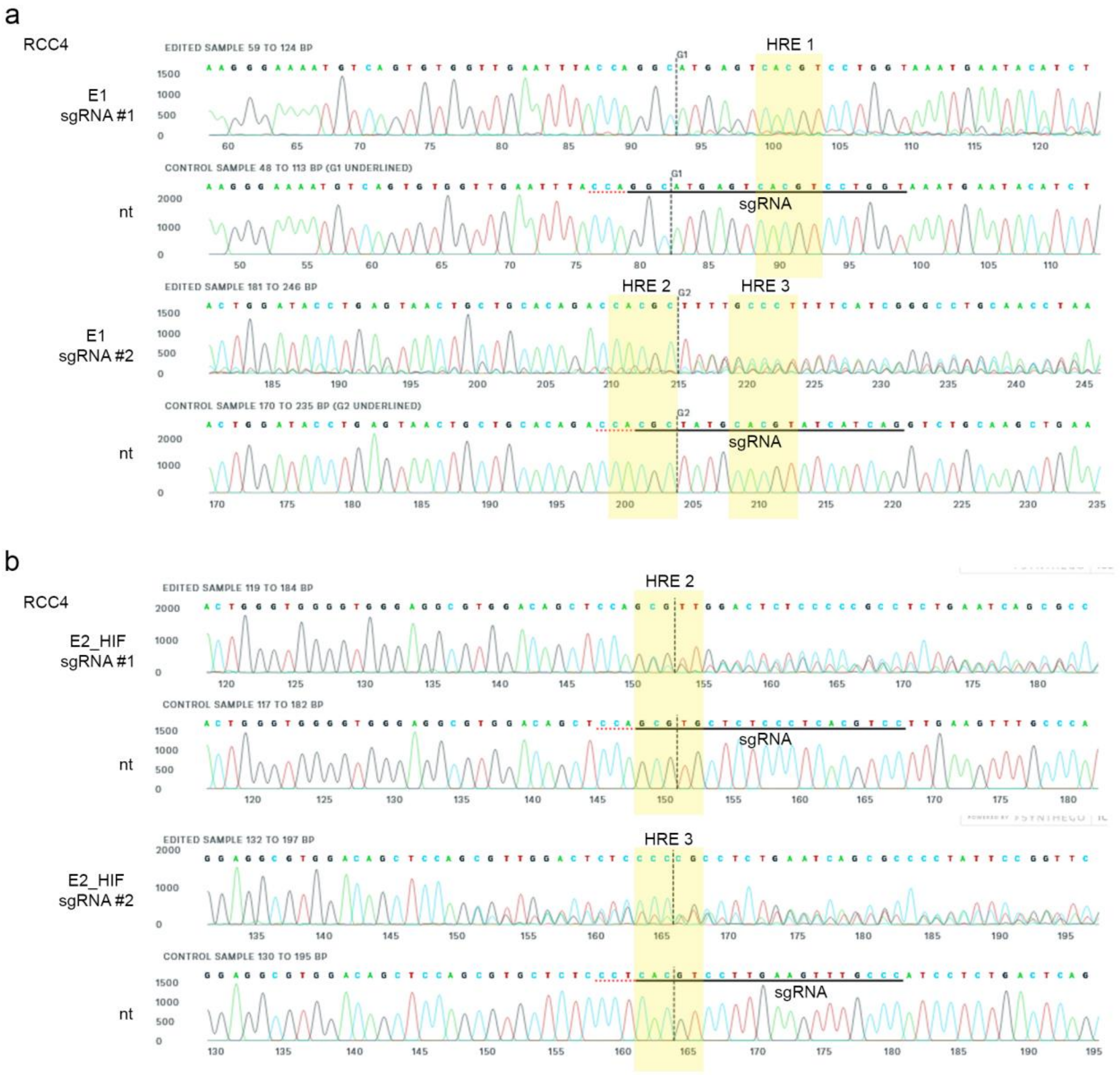

Supplementary Figure 23: "Inference of CRISPR Edits" (ICE) software analysis of HIF binding site knock-out at *EPAS1*-enhancers 1 and 2 in RCC4 cell pools. a) Sanger sequencing tracks of control and edited DNA isolated from RCC4 cell pools treated with sgRNAs targeting the hypoxia-responsive elements 1-3 (HRE 1-3) at *EPAS1*-enhancer 1 (E1 sgRNA #1 and #2) or non-targeting control sgRNAs (nt). The target region of the sgRNAs and the transcription factor motif are highlighted. **b)** Sanger sequencing tracks of control and edited DNA isolated from RCC4 cell pools treated with sgRNAs targeting the hypoxia-responsive elements 2 and 3 (HRE 2 and 3) at *EPAS1*-enhancer 2 (E2_HIF sgRNA #1 and #2) or non-targeting control sgRNAs (nt). The target region of the sgRNA and the transcription factor motif are highlighted.

Supplementary Figure 24

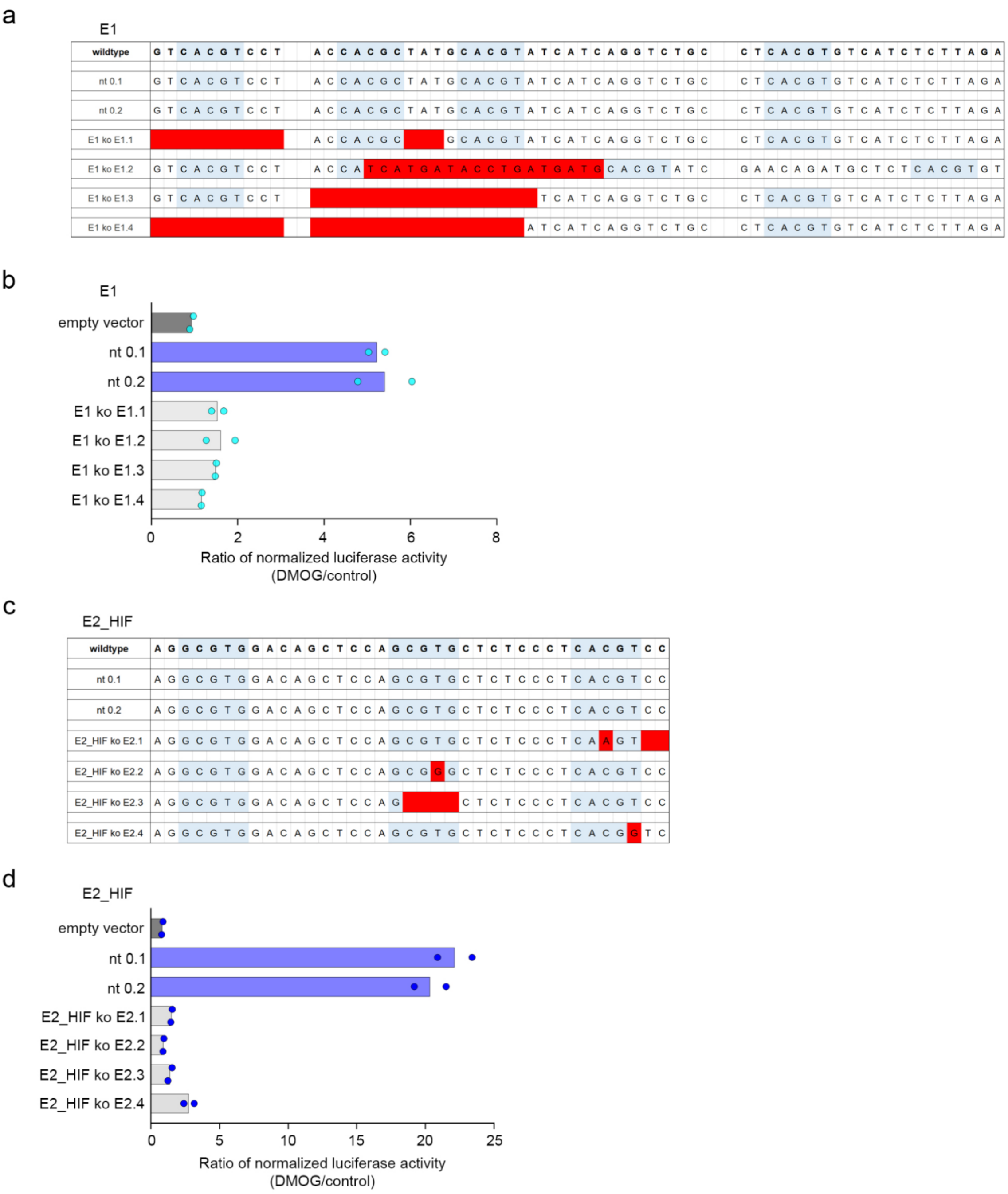

Supplementary Figure 24: Reporter gene assay using *EPAS1*-enhancer E1 and E2 sequences generated by CRISPR/Cas9 mutation. a) Sanger sequencing reveals defective HREs in *EPAS1*-enhancer E1 knock-out single clones of 786-0 cells (E1 ko E1.1 – E1 ko E1.4). Single clones of cells treated with non-targeting control sgRNA harbor intact wildtype sequences (nt 0.1 and nt 0.2). Mutations are highlighted in red. **b)** Reporter assay using sequences shown in a). Kelly cells were transfected with a pGL3-promoter vector alone or constructs containing intact or defective *EPAS1*-enhancer 1 sequences. Cells were exposed to 1 mM DMOG or control conditions and reporter activity was measured after 16h of stimulation. Values of luciferase activity were first normalized to activity of co-transfected β-galactosidase and subsequently to the activity of the respective control condition without HIF-stabilization. Bars indicate mean of two independent experiments. **c)** Sanger sequencing shows defective hypoxia-responsive elements in E2_HIF knock-out single clones of 786-0 cells (E2_HIF ko E2.1 – E2_HIF ko E2.4). Single clones of cells treated with non-targeting control sgRNA exhibit intact wildtype sequences (nt 0.1 and nt 0.2). Mutations are highlighted in red. **d)** Reporter assay using sequences shown in c). Kelly cells were transfected with a pGL3-promoter vector alone or with constructs containing intact or defective *EPAS1*-enhancer 2 sequences. Cells were exposed to 1 mM DMOG or control conditions and reporter activity was measured after 16h of stimulation. Values of luciferase activity were first normalized to activity of co-transfected β-galactosidase and subsequently to the activity of the respective control condition without HIF-stabilization. Bars indicate mean of two independent experiments.

Supplementary Figure 25

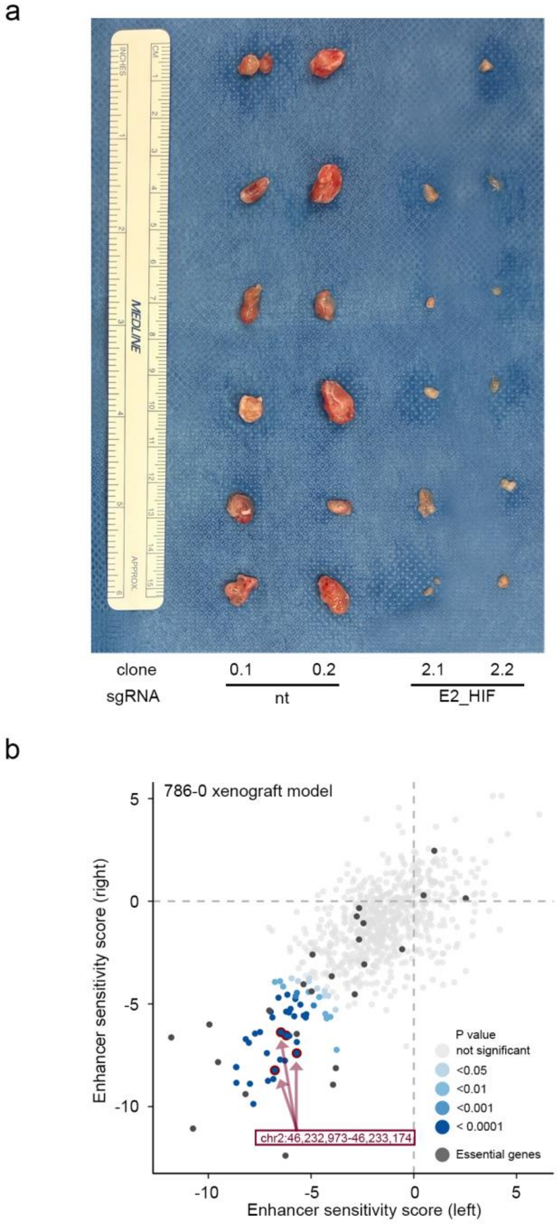

Supplementary Figure 25: *EPAS1*-enhancer 2 affects tumor growth in an *in vivo* mouse model. a) Xenograft tumor assay in NOD/SCID-gamma mice using *EPAS1*-enhancer 2 defective (E2_HIF ko) or intact (nt) 786-0 single clones of cells. Size of xenograft tumors explanted at week 10. **b)** CRISPRi screen from Patel et al.[12] depicting sensitivity scores for individual constructs targeting HIF-2α-bound enhancers in 786-M1A cell xenografts (n=30 tumors, 2 groups: right versus left mouse flank). sgRNAs directed against *EPAS1*-enhancer 2 are highlighted. Empirical p-values of one-sided enrichment test based on 10,000 permutations as determined by Patel et al.

Supplementary Figure 26

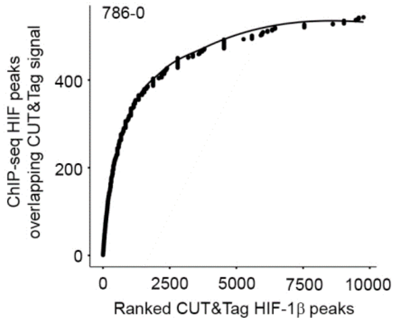

Suppementary Figure 26: Validation of CUT&Tag-seq results. To evaluate HIF-1β binding sites determined by CUT&Tag-seq in 786-0 clones of cells treated with non-targeting control guides, we correlated these peaks with 805 ChIP-seq validated HIF-1β DNA binding sites in 786-0 cells[13]. CUT&Tag-seq signals were ranked according to the -log10 (q-value). 543 peaks overlapped and top ranked CUT&Tag-seq peaks showed remarkable overlap with the ChIP-seq validated HIF-1β binding sites.

Supplementary Information References

1. Chang, K., *et al.* The Cancer Genome Atlas Pan-Cancer analysis project. *Nature Genetics* **45**, 1113-1120 (2013).
2. Salama, R., *et al.* Heterogeneous Effects of Direct Hypoxia Pathway Activation in Kidney Cancer. *PLoS One* **10**, e0134645 (2015).
3. Yao, X., *et al.* VHL Deficiency Drives Enhancer Activation of Oncogenes in Clear Cell Renal Cell Carcinoma. *Cancer Discov* **7**, 1284-1305 (2017).
4. Smythies, J.A., *et al.* Inherent DNA-binding specificities of the HIF-1alpha and HIF-2alpha transcription factors in chromatin. *EMBO Rep* **20**(2019).
5. Corces, M.R., *et al.* The chromatin accessibility landscape of primary human cancers. *Science* **362**(2018).
6. Schoenfeld, D.A., *et al.* Loss of PBRM1 Alters Promoter Histone Modifications and Activates ALDH1A1 to Drive Renal Cell Carcinoma. *Mol Cancer Res* **20**, 1193-1207 (2022).
7. Zou, Y., *et al.* A GPX4-dependent cancer cell state underlies the clear-cell morphology and confers sensitivity to ferroptosis. *Nat Commun* **10**, 1617 (2019).
8. Schmid, V., *et al.* Co-incidence of RCC-susceptibility polymorphisms with HIF cis-acting sequences supports a pathway tuning model of cancer. *Sci Rep* **9**, 18768 (2019).
9. Tiana, M., *et al.* The SIN3A histone deacetylase complex is required for a complete transcriptional response to hypoxia. *Nucleic Acids Res* **46**, 120-133 (2018).
10. Meuleman, W., *et al.* Index and biological spectrum of human DNase I hypersensitive sites. *Nature* **584**, 244-251 (2020).
11. Han, S.S., *et al.* The chromosome 2p21 region harbors a complex genetic architecture for association with risk for renal cell carcinoma. *Human Molecular Genetics* **21**, 1190-1200 (2012).
12. Patel, S.A., *et al.* The renal lineage factor PAX8 controls oncogenic signalling in kidney cancer. *Nature* **606**, 999-1006 (2022).
13. Schodel, J., *et al.* Common genetic variants at the 11q13.3 renal cancer susceptibility locus influence binding of HIF to an enhancer of cyclin D1 expression. *Nat Genet* **44**, 420-425, S421-422 (2012).
