# Supplementary material for: HIF sustain a transcriptional regulatory circuit of EPAS1 expression in renal clear cell carcinoma
Source: Nat Commun. 2026 Feb 19;17:1764. doi: 10.1038/s41467-026-68576-0 (PMC12921326; doi:10.1038/s41467-026-68576-0)
Supplement: Supplementary file 2 — Description of Additional [file 41467_2026_68576_MOESM2_ESM.pdf]

Supplementary Data Legends

Supplementary Data 1: DEG analysis of ccRCC cells versus PTC. For the analysis of differentially expressed genes (DEG) in ccRCC tumor cells versus primary tubule cells (PTC) RNA-seq samples of three different patients each sequenced in duplicates were examined. DEG analysis was performed using DESeq2. Adjusted p-values were calculated using the Benjamini–Hochberg method.

Supplementary Data 2: Coordinates of Meta-HIF ChIP-seq data set. The Meta-HIF ChIP-seq data set integrates published sequencing data of HIF-1α, HIF-1β and HIF-2α ChIP experiments in RCC4, 786-0 and primary ccRCC cells[1-3].

Supplementary Data 3: DOR analysis of ccRCC cells versus PTC. For the analysis of differentially open regions (DOR) in ccRCC tumor cells versus primary tubule cells (PTC) ATAC samples from three different patients each sequenced in duplicates were examined. DOR analysis was performed using DESeq2. Adjusted p-values were calculated using the Benjamini–Hochberg method. Overlap with positive H3K27ac ChIP-seq signal based on ChIP-seq experiments in three different patients is indicated for each ATAC-defined region.

Supplementary Data 4: ABC-predicted interactions for PTC, ccRCC and 786-0 cells. The activity-by-contact (ABC) model was applied to predict interactions between enhancer sites and putative target genes in primary tubule cells (PTC), primary ccRCC and 786-0 cells. Inputs for the individual-specific analysis were bam files for ATAC-seq and H3K27ac ChIP-seq as well as TPM-normalized RNA-seq data from corresponding PTC and ccRCC cells of three different patients. The ABC analysis for 786-0 cells is based on published ATAC-seq, H3K27ac ChIP-seq and RNA-seq data[2,4,5].

Supplementary Data 5: DEG analysis of U-87 cells treated with DMOG versus control condition. Analysis of differentially expressed genes (DEG) for U-87 cells treated with 1 mM DMOG for 16h or cultured under control conditions was performed using DESeq2. Adjusted p-values were calculated using the Benjamini–Hochberg method. Technical duplicates per condition were examined.

Supplementary Data 6: DOR analysis of single 786-0 cell clones with HNF-1β ko versus nt. Analysis of differentially open regions (DOR) was performed using DESeq2. Adjusted p-values were calculated using the Benjamini–Hochberg method. For the detection of DOR between HNF-1β knock-out (ko) cells versus cells treated with non-targeting (nt) control sgRNA, sequencing duplicates of two single clones of cells were used per condition.

Supplementary Data 7: DOR analysis of single 786-0 cell clones with PAX8 ko versus nt. Analysis of differentially open regions (DOR) was performed using DESeq2. Adjusted p-values were calculated using the Benjamini–Hochberg method. For the detection of DOR between PAX8 knock-out (ko) cells

versus cells treated with non-targeting (nt) control sgRNA, ATAC-seq data of two single clones of cells each sequenced in duplicates were used per condition.

Supplementary Data 8: DEG analysis of 786-0 cell pool with E2_HIF ko versus nt. Analysis of differentially expressed genes (DEG) was performed using DESeq2. Adjusted p-values were calculated using the Benjamini–Hochberg method. For the detection of DEG between the E2_HIF knock-out (ko) cell population versus the 786-0 cell population treated with non-targeting (nt) control sgRNA, RNA-seq data each sequenced in duplicates were used per condition.

Supplementary Data 9: DEG analysis of RCC4 cell pool with E2_HIF ko versus nt. Analysis of differentially expressed genes (DEG) was performed using DESeq2. Adjusted p-values were calculated using the Benjamini–Hochberg method. For the detection of DEG between the E2_HIF knock-out (ko) cell population versus the RCC4 cell population treated with non-targeting (nt) control sgRNA, RNA-seq data each sequenced in duplicates were used per condition.

Supplementary Data 10: DEG analysis of single cell 786-0 clones with E2_HIF ko versus nt. Analysis of differentially expressed genes (DEG) was performed using DESeq2. Adjusted p-values were calculated using the Benjamini–Hochberg method. For the detection of DEG between E2_HIF knock-out (ko) cells versus cells treated with non-targeting (nt) control sgRNA, RNA-seq data of two single clones of cells each sequenced in duplicates were used per condition.

Supplementary Data 11: sgRNA and oligonucleotide sequences. Sequences of sgRNA and primers used in this study.

Supplementary Data 12: Densitometry of immunoblots. Quantification of HIF-2α signals detected by immunoblotting normalized to β-Actin.

Supplementary Data 13: QC RNA-seq. Quality control (QC) metrics for RNA-seq data generated in this study. RIN = RNA integrity number.

Supplementary Data 14: QC ATAC-seq. Quality control (QC) metrics for ATAC-seq data generated in this study. FRiP = Fraction of Reads in Peaks.

Supplementary Data 15: QC ChIP-seq. Quality control (QC) metrics for ChIP-seq data generated in this study.

Supplementary References

1. Salama, R.*, et al.* Heterogeneous Effects of Direct Hypoxia Pathway Activation in Kidney Cancer. *PLoS One* **10**, e0134645 (2015).
2. Yao, X.*, et al.* VHL Deficiency Drives Enhancer Activation of Oncogenes in Clear Cell Renal Cell Carcinoma. *Cancer Discov* **7**, 1284-1305 (2017).
3. Smythies, J.A.*, et al.* Inherent DNA-binding specificities of the HIF-1alpha and HIF-2alpha transcription factors in chromatin. *EMBO Rep* **20**(2019).
4. Schoenfeld, D.A.*, et al.* Loss of PBRM1 Alters Promoter Histone Modifications and Activates ALDH1A1 to Drive Renal Cell Carcinoma. *Mol Cancer Res* **20**, 1193-1207 (2022).
5. Zou, Y.*, et al.* A GPX4-dependent cancer cell state underlies the clear-cell morphology and confers sensitivity to ferroptosis. *Nat Commun* **10**, 1617 (2019).
