# Supplementary material for: HIF sustain a transcriptional regulatory circuit of EPAS1 expression in renal clear cell carcinoma
Source: Nat Commun. 2026 Feb 19;17:1764. doi: 10.1038/s41467-026-68576-0 (PMC12921326; doi:10.1038/s41467-026-68576-0)
Supplement: Supplementary file 4 — Reporting Summary [file 41467_2026_68576_MOESM4_ESM.pdf]

nature portfolio

Corresponding author(s): Johannes Schödel

Last updated by author(s): Dec 17, 2025

Reporting Summary

Nature Portfolio wishes to improve the reproducibility of the work that we publish. This form provides structure for consistency and transparency in reporting. For further information on Nature Portfolio policies, see our Editorial Policies and the Editorial Policy Checklist.

Statistics

For all statistical analyses, confirm that the following items are present in the figure legend, table legend, main text, or Methods section.

n/a	Confirmed	
☐	☒	The exact sample size (*n*) for each experimental group/condition, given as a discrete number and unit of measurement
☐	☒	A statement on whether measurements were taken from distinct samples or whether the same sample was measured repeatedly
☐	☒	The statistical test(s) used AND whether they are one- or two-sided<br>*Only common tests should be described solely by name; describe more complex techniques in the Methods section.*
☒	☐	A description of all covariates tested
☐	☒	A description of any assumptions or corrections, such as tests of normality and adjustment for multiple comparisons
☐	☒	A full description of the statistical parameters including central tendency (e.g. means) or other basic estimates (e.g. regression coefficient) AND variation (e.g. standard deviation) or associated estimates of uncertainty (e.g. confidence intervals)
☐	☒	For null hypothesis testing, the test statistic (e.g. *F*, *t*, *r*) with confidence intervals, effect sizes, degrees of freedom and *P* value noted<br>*Give P values as exact values whenever suitable.*
☒	☐	For Bayesian analysis, information on the choice of priors and Markov chain Monte Carlo settings
☒	☐	For hierarchical and complex designs, identification of the appropriate level for tests and full reporting of outcomes
☐	☒	Estimates of effect sizes (e.g. Cohen's *d*, Pearson's *r*), indicating how they were calculated

Our web collection on statistics for biologists contains articles on many of the points above.

Software and code

Policy information about availability of computer code

Data collection

Quantitative RT–PCR data was generated on a StepOnePlus Real-Time PCR cycler using integrated software (Applied Biosystems, ThermoFisher Scientific, Waltham, MA, USA).
Immunoblot signal detection was performed on an Amersham Imager 600 using integrated software (GE Healthcare, Amersham, UK).
DNA concentration of libraries and fragment size distribution were determined by electrophoresis using the Agilent 2100 Bioanalyzer using integrated software (Agilent, Santa Clara, CA, USA).
Images of matrigel experiments were taken using the Zeiss Primo Vert microscope and Zeiss Axiocam 105 camera with integrated software (Zeiss Microscopy GmbH, Oberkochen, Germany).
Staining was visualized with a Leica DM 6000B microscope using integrated software (Leica, Wetzlar, Germany).
A Caliper (Carl Roth GmbH, Germany) was used to measure tumor size and a precision balance (Kern und Sohn GmbH, Germany) to measure tumor weight in the tumor xenograft experiments.

Data analysis

- FastQC (v0.11.8) was used for quality control of NGS data.
- STAR (v2.7.10a) was used for alignment of RNA reads.
- ATAC-seq and CUT&Tag-seq reads were aligned to the reference genome hg38 using Bowtie2 (2.3.4.1).
- Burrows-Wheeler Aligner (BWA-mem; 0.7.17-r1188) was applied for alignment of ChIP-seq reads.
- feature-counts software (1.6.1) was used for creation of a mapped RNA reads table.
- DESeq2 package (1.32.0) was applied for logarithmic transformation of NGS data and analysis of differentially expressed genes. Version 1.28.1 was used for detection of differentially accessible regions.
- biomaRT (2.48.3) was used for gene annotation.
- Trim Galore (0.6.6) was applied for adapter trimming of ATAC-seq and ChIP-seq reads.

• SAMtools (1.8) was used for removal of DNA reads with a Mapping Quality (MAPQ) below 30 and those mapped to ChrM. Technical or biological (as indicated) ChIP-, CUT&Tag- and ATAC-seq duplicates were merged using SAMtools.
• Removal of PCR-duplicates was performed by Picard (v2.25.2).
• GenomicRanges (v1.46.1) and regioneR (v1.26.1) were used for removal of ENCODE hg38 blacklisted regions and chromosome Y as well as generation of reference peak set ("reduce" function).
• MACS2 (v2.2.7.1) was used to perform peak calling.
• DiffBind (v. 3.4.11) was applied to analyse ChIP-seq or CUT&Tag-seq H3K27ac peaks and to establish a consensus peak set.
• Peak signal intensity was analyzed using the computeMatrix function and results were visualized with the plotProfile function of the deepTools suite (v. 3.5.1).
• Activity by Contact (ABC) Model of Enhancer-Gene Specifity29 (v1.0) was applied to predict enhancers that regulate EPAS1 expression in primary tubule and ccRCC cells.
• BamCoverage (v3.3.2) was applied for generating normalized bigWig tracks.
• bigWig signals of target regions were extracted using the import function of the rtracklayer package (v1.54.0).
• Wiggletools (v1.2.11) normalized bedGraph files.
• bedGraphToBigWig tool was used for conversion of bedGraph files to bigWig formats.
• HOMER2 (v4.9.1) was used for exploration of motifs in DNA target regions.
• R Package ggpubr (v0.4.0) allowed for visualization of motif enrichment results.
• GenomicRanges (v1.46.1) was used for overlapping CUT&Tag-signals. computeMatrix allowed for quantification of the peak signal intensity which was visualized with the plotProfile function of deepTools.
• The above-mentioned R packages were conducted in R version 4.1.1.
• CrossMap (v0.6.1) (Python v3.5) was used for liftover of published Capture-C data and ChIP-seq data from hg19 to hg38.
• Processed CRISPRi screening data including the analysis script from Patel et al. (doi: 10.1038/s41586-022-04809-8) were downloaded from https://zenodo.org/record/6335339.
• SnATAC-seq co-accessibility analysis was conducted in R version 4.2.2. using R packages Signac (version 1.12.0), GenomicRanges (version 1.50.2), Seurat (version 5.0.1) and Cicero (version 1.3.9).
• Differentially active enhancer regions in ccRCC tumors from Yao et al. (doi: 10.1158/2159-8290.CD-17-0375) were plotted using the R package EnhancedVolcano (version 1.22.0) in R version 4.2.2.
• All statistical analyses excluding sequencing data were performed using GraphPadPrism Version 9.0.2 (GraphPad Software Inc., San Diego, CA, USA).

For manuscripts utilizing custom algorithms or software that are central to the research but not yet described in published literature, software must be made available to editors and reviewers. We strongly encourage code deposition in a community repository (e.g. GitHub). See the Nature Portfolio guidelines for submitting code & software for further information.

Data

Policy information about availability of data

All manuscripts must include a data availability statement. This statement should provide the following information, where applicable:

- Accession codes, unique identifiers, or web links for publicly available datasets
- A description of any restrictions on data availability
- For clinical datasets or third party data, please ensure that the statement adheres to our policy

ATAC-seq, ChIP-seq, CUT&Tag-seq, and RNA-seq data generated for this project have been deposited in the GEO database under accession number GSE256001 (https://www.ncbi.nlm.nih.gov/geo/query/acc.cgi?acc=GSE256001). Access to unprocessed raw data of primary cells is restricted to protect the privacy and intent of research participants consistent with the written informed consent agreements provided by individual research participants.

The following publicly available data sets were used:
GEO Database:
Capture-C data in 786-0: GSE130988, https://www.ncbi.nlm.nih.gov/geo/query/acc.cgi?acc=GSE130988;
HIF-1β and HIF-2α ChIP-seq data in 786-0: GSE67237, https://www.ncbi.nlm.nih.gov/geo/query/acc.cgi?acc=GSE67237;
HIF-1β ChIP-seq data from 786-0: GSE34871, https://www.ncbi.nlm.nih.gov/geo/query/acc.cgi?acc=GSE34871;
HIF-ChIP-seq in ccRCC cells and H3K27ac ChIP-seq in normal kidney and tumor tissue as well as 786-0 cells: GSE86095, https://www.ncbi.nlm.nih.gov/geo/query/acc.cgi?acc=GSE86095;
ATAC-seq in 786-0 cells: GSE102807, https://www.ncbi.nlm.nih.gov/geo/query/acc.cgi?acc=GSE102807;
HIF-1β ChIP in T47D, A549, HCT116 and PC3 cells: GSE130989, https://www.ncbi.nlm.nih.gov/geo/query/acc.cgi?acc=GSE130989;
HIF-1β ChIP in HepG2, HKC-8 and RCC4: GSE120885, https://www.ncbi.nlm.nih.gov/geo/query/acc.cgi?acc=GSE120885;
HIF-1β ChIP in HUVEC: GSE89836, https://www.ncbi.nlm.nih.gov/geo/query/acc.cgi?acc=GSE89836;
HIF-1β ChIP in Hela: GSE159128, https://www.ncbi.nlm.nih.gov/geo/query/acc.cgi?acc=GSE159128;
HIF-1β ChIP in PTC #4: GSE101063, https://www.ncbi.nlm.nih.gov/geo/query/acc.cgi?acc=GSE101063
RNA-seq in 786-0: GSE115389, https://www.ncbi.nlm.nih.gov/geo/query/acc.cgi?acc=GSE115389;
RNA-seq in 786-0-xenografts with or without PT2385 treatment: GSE153711, https://www.ncbi.nlm.nih.gov/geo/query/acc.cgi?acc=GSE153711;
snATAC-seq data and metadata: GSE240822, https://www.ncbi.nlm.nih.gov/geo/query/acc.cgi?acc=GSE240822.

KIRC regulatory elements from TCGA were downloaded from https://gdc.cancer.gov/about-data/publications/ATACseq-AWG (access 24.03.2021). RSEM-normalized RNA-seq data from TCGA were downloaded using RTCGA.rnaseq (v.0.1-3) (access 28.05.2021). Data for significantly hypomethylated CpGs in KIRC were accessed via http://www.bioinfo-zs.com/smartapp/ (28.11.23). TCGA data for the methylation status of individual CpGs at the PRKCE-EPAS1 locus in the KIRC dataset (normal and tumor tissue) were downloaded from http://maplab.imppc.org/wanderer/ (access 12.04.2024). Copy number alterations and corresponding RNA-seq data from the TCGA PanCancer Atlas were downloaded via the cBioPortal (https://www.cbioportal.org/, original data: https://gdc.cancer.gov/about-data/publications/pancanatlas, access 20.07.2023). SNPs with cis-eQTLs for KIRC were extracted from http://gong_lab.hzau.edu.cn/PancanQTL. Data from 733 biosamples were published by Meuleman et al. and were accessed via a public server (https://index.altius.org/, access 03.08.2023). Sequencing data and analysis form CRISPRi screen for oncogenic-HIF-2-bound enhancer sites in 786-M1A cells were downloaded from https://zenodo.org/records/6335339 (access 06.02.2023). Transcription factor motifs were downloaded from the JASPAR 2024 database (access 03.12.2024).
Access to the restricted TCGA genotyping data for the KIRC, KICH and KIRP cohorts presented in the current publication was granted via phs000178 at the dbGaP web site. These data were generated by the TCGA Research Network and can be accessed via https://www.cancer.gov/tcga (access KIRC data: 31.10.2024, access

KICH and KIRP data: 11.12.2024).

Research involving human participants, their data, or biological material

Policy information about studies with human participants or human data. See also policy information about sex, gender (identity/presentation), and sexual orientation and race, ethnicity and racism.

Reporting on sex and gender	Tissue samples used for cell isolation were taken from nephrectomies from anonymised donors and assigned to a code number. Neither sex nor gender were retrospectively enquired. Isolated cells were sequentially included in the study according to the availability of nephrectomy specimens. We did not conduct sex- or gender-specific analyses, as this was not the focus of the study.
Reporting on race, ethnicity, or other socially relevant groupings	Human specimens were collected with an assigned code number without knowledge of ethnicity of the donor. The majority of patients undergoing tumor nephrectomy at the Urology Department of the Uniklinikum Erlangen are of Caucasian ethnicity.
Population characteristics	In Europe, men are more frequently affected by kidney cancer with an incidence of approx. 26/100,000 than women with an incidence of approx. 12/100,000. The average age of onset is between 65 and 70 years for men and over 70 years for women. 105 kidney specimens used for isolation of primary tubular cells and 34 ccRCC specimens for isolation of tumor cells were consecutively acquired from tumor nephrectomies performed in the Urology Department. ccRCC specimens included in this study were only selected based on their cancer entity according to the diagnosis of an expert pathologist. Since no further specific inclusion or exclusion criteria were applied these specimens should mirror the above-mentioned population characteristics.
Recruitment	Adult study participants were recruited through the Comprehensive Cancer Center Erlangen-EMN (CCC) at the Uniklinikum Erlangen. Anonymised tumor and normal kidney samples were examined and diagnosed by an expert pathologist. Specimens were sequentially included. There was no potential for self-selection bias.
Ethics oversight	Each patient gave informed consent and the local ethics committee at the University of Erlangen-Nürnberg approved use of the tissue (329_16B; 542_20Bc). Specimens were collected in accordance with the World Medical Association Declaration of Helsinki.

Note that full information on the approval of the study protocol must also be provided in the manuscript.

Field-specific reporting

Please select the one below that is the best fit for your research. If you are not sure, read the appropriate sections before making your selection.

☒ Life sciences ☐ Behavioural & social sciences ☐ Ecological, evolutionary & environmental sciences

For a reference copy of the document with all sections, see nature.com/documents/nr-reporting-summary-flat.pdf

Life sciences study design

All studies must disclose on these points even when the disclosure is negative.

Sample size	Patient material was collected consecutively. Sample size was therefore determined by the availability of human specimens. RNA expression analysis in tissue lysates from renal tumors or corresponding normal kidney tissue from the Erlangen RCC cohort include 114 clear cell renal carcinoma, 16 papillary renal cell carcinoma and 11 chromophobe renal cell carcinoma samples reflecting the known distribution of RCC subtypes. 105 kidney specimens for isolation of primary cells and 34 ccRCC specimens for isolation of tumor cells were sequentially acquired from tumor nephrectomies performed in the Urology Department.
Data exclusions	Specimens from the Erlangen RCC cohort: 28 out of 142 clear cell renal cell carinoma, 2 out of 18 papillary and 3 out of 13 chromophobe renal cancer samples were excluded from further analysis due to insufficient RNA quality as determined by expression of housekeeping genes.<br>NGS data: Reads with a Mapping Quality (MAPQ) below 30 and those mapped to ChrM were removed using SAMtools (1.8). ENCODE hg38 blacklisted regions were excluded.
Replication	Results acquired in primary human samples were validated in human tissue from the Erlangen RCC cohort and in isolated ccRCC cells.<br>ATAC-seq, RNA-seq and ChIP-seq were performed in isolated primary tubule and corresponding ccRCC cells from 3 different individuals.<br>Transcriptomic and epigenetic results were correlated with published datasets from the TCGA KIRC-cohort.<br>Enhancer and gene knock-out experiments were conducted in 2 different RCC cell lines (786-0 and RCC-4) to validate results.<br>RNA-seq and ATAC-seq of primary cells as well as cell lines were performed in technical duplicates according to ENCODE standards. RNA-seq, ATAC-seq and CUT&Tag-seq experiments in single clones of cells (conditions: intact versus defective enhancer site, non-targeting control versus HIF-1b ko or HNF-1b ko or PAX8 ko) were replicated in at least two different clones.<br>Exceptions from the abovementioned standards were ChIP-seq and CUT&Tag experiments with primary human cells due to restricted specimen availability.<br>Quantitative PCR experiments in cell lines include 3 - 6 biological replicates. qPCR expression values were determined in duplicates per sample. For immunoblot analysis we performed at least 2 independent experiments. Representative blots are shown.<br>For the xenograft study 2 different single clones of 786-0 cells per condition (intact versus defective enhancer site) were analysed.

Randomization	The study was observational and did not involve allocating patients into intervention or treatment groups. Thus, randomization was not relevant for study design.
Blinding	The part of the study that refers to human data was observational and did not involve allocating patients into intervention or treatment groups. Thus, blinding was not relevant for this part of the study. For the murine xenograft tumor model NOD/SCID-gamma mice were injected with single clones of 786-0 cells (intact versus defective enhancer site) in a randomized, non-blinded manner. The tumor growth was monitored by an investigator blinded for the different conditions.

Reporting for specific materials, systems and methods

We require information from authors about some types of materials, experimental systems and methods used in many studies. Here, indicate whether each material, system or method listed is relevant to your study. If you are not sure if a list item applies to your research, read the appropriate section before selecting a response.

Materials & experimental systems

n/a	Involved in the study	
☐	☒	Antibodies
☐	☒	Eukaryotic cell lines
☒	☐	Palaeontology and archaeology
☐	☒	Animals and other organisms
☒	☐	Clinical data
☒	☐	Dual use research of concern
☒	☐	Plants

Methods

n/a	Involved in the study	
☐	☒	ChIP-seq
☒	☐	Flow cytometry
☒	☐	MRI-based neuroimaging

Antibodies

Antibodies used

Primary antibodies used for WB:
rabbit anti-HIF-1α (Cay10006421, Cayman Chemicals, Ann Arbor, MI, USA), dilution: 1:1,000
mouse anti-HIF-1α (610959, BD Biosciences, New Jersey, USA), dilution: 1:500
goat anti-HIF-2α (AF2997, R&D Systems, Minneapolis, USA), dilution: 1:250
rabbit anti-HIF-2α (A700-003, Bethyl, Waltham, USA), dilution: 1:1,000
rabbit anti-HIF-1β (NB100-110, Novus Biologicals, Biotechne, Wiesbaden, Germany), dilution: 1:1,000
rat anti-hemagglutinin (HA) (11867423001, Roche Diagnostics GmbH, Mannheim, Germany), dilution: 1:1,000
rabbit anti-VHL (68547, Cell Signaling Technology, Danvers, USA), dilution: 1:1,000
rabbit anti-PAX8 (10336-1-AP, Proteintech, Planegg-Martinsried, Germany), dilution: 1:5,000
rabbit anti-HNF-1β (HPA002083, Atlas Antibodies, Sigma Aldrich, St. Louis, MO, USA), dilution 1:2,000
mouse anti–β-actin-peroxidase (A3854, Sigma Aldrich, St. Louis, MO, USA), dilution 1:60,000

Secondary antibodies for WB:
Horseradish peroxidase-conjugated anti-rabbit (P0399, swine polyclonal, Agilent Technologies, Santa Clara, CA, USA), dilution: 1:2,500
anti-goat (P0449, rabbit polyclonal, Agilent Technologies, Santa Clara, CA, USA), dilution 1:2,500
anti-rat (Jackson ImmunoResearch, Ely, UK), dilution 1:2,500

Chromatin Immunoprecipitation:
For immunoprecipitations, 70 µg of chromatin and 3-6µl of these antibodies were used:
rabbit anti-HIF-1β (NB100-110, Novus Biologicals, Biotechne, Wiesbaden, Germany)
rabbit anti-H3K27ac (ab4729, Abcam, Cambridge, UK)
rabbit anti-HNF-1β (HPA002083, Atlas Antibodies, Sigma Aldrich)

Primary antibodies used for CUT&Tag:
rabbit anti-HIF-1α (Cay10006421, Cayman Chemicals, Ann Arbor, MI, USA), dilution: 1:50
rabbit anti-HIF-1β antibody (5537S, Cell Signaling Technology, Danvers, MA, USA), dilution: 1:50
rabbit anti-HIF-1β (NB100-110, Novus Biologicals, Biotechne, Wiesbaden, Germany), dilution: 1:50
rabbit anti-HNF-1β (HPA002083, Atlas Antibodies, Sigma Aldrich, St. Louis, MO, USA), dilution: 1:50
rabbit anti-H3K27ac antibody (ab177178, Abcam, Cambridge, UK), dilution: 1:50

Secondary antibody for CUT&Tag:
guinea Pig anti-Rabbit IgG antibody (ABIN101961, Antibodies-Online, Aachen, Germany), dilution: 1:50

Validation

rabbit anti-HIF-1α (Cay10006421, Cayman Chemicals) was validated for use in WB in previous work (doi.org/10.1074/jbc.RA119.009827, doi.org/10.1016/j.jbc.2022.101699) by using siRNA against HIF-1α in different cell lines.
mouse anti-HIF-1α (610959, BD Biosciences) was validated by manufacturer for WB and IHC.
anti-HIF-2α (AF2997, R&D Systems) was validated for WB and IHC by manufacturer. Specifity was confirmed by WB of protein lysates in cells treated with siRNA against HIF-2α (doi.org/10.1016/j.jbc.2022.101699).
anti-HIF-2α (A700-003, Bethyl) was validated by manufacturer for WB, IHC, IF, IP and ChIP-seq. Antibody was validated in-house for these applications using CRISPR/Cas edited knock-out and siRNA-mediated knockdown experiments (data not shown).
rabbit anti-HIF-1β (NB100-110, Novus Biologicals) was extensively validated for ICC, IHC, IP WB and ChIP-seq by manufacturer and in

previous work (doi: 10.1038/ng.2204, doi: 10.1038/ncomms13183).
anti-hemagglutinin (HA) (11867423001, Roche) was validated by manufacturer for WB and ELISA and in-house using a RCC4/i.VHL-HA ccRCC cell line which contains a doxycycline inducible HA-tagged VHL expression DNA element (see manuscript, Fig. 3c).
rabbit anti-VHL (68547, Cell Signaling Technology) was validated by manufacturer and in-house for WB using VHL-null and VHL-reexpressing renal cancer cell lines.
anti-PAX8 (10336-1-AP, Proteintech) was validated by manufacturer for WB, IP, IHC and IF . We validated this antibody in CRISPR/Cas-mediated PAX8 knock-out in 786-0 cells (see manuscript Fig. 5d).
anti-HNF-1β (HPA002083, Atlas Antibodies) was validated by manufacturer for ICC, IHC and WB. We validated this antibody in CRISPR/Cas-mediated HNF-1β knock-out in 786-0 cells (see manuscript Fig. 5c). ChIP-seq with this antibody in ccRCC cell lines OS-LM1 and 786-M1A was previously published (doi: 10.1038/s41586-022-04809-8).
anti-H3K27ac (ab4729, Abcam, Cambridge, UK) was validated by manufacturer for ChIP-seq and extensively validated in published literature (e.g. doi: 10.1038/ng.2204, doi: 10.1038/ncomms13183).

Eukaryotic cell lines

Policy information about cell lines and Sex and Gender in Research

Cell line source(s)	Primary cells were isolated from ccRCC tissue and healthy human kidney cortical tissue from patients undergoing tumor nephrectomy at the Uniklinikum Erlangen. Sex was not determined. 786-0 and Caki-1 cells were purchased form ATCC. Kelly cells were a gift from C. Warnecke and U-87 cells were a gift from F. Müller, Erlangen, Germany. RCC4 and RCC4/i-VHL-HA cells were from P. Ratcliffe, Oxford, UK.
Authentication	Cell lines were authenticated by STR profiling (Gene Print 10 System by Promega).
Mycoplasma contamination	All cell lines used were tested negative for mycoplamsa contamination.
Commonly misidentified lines (See ICLAC register)	None of the cell lines used in this study were among the misidentified lines.

Animals and other research organisms

Policy information about studies involving animals; ARRIVE guidelines recommended for reporting animal research, and Sex and Gender in Research

Laboratory animals	NOD/SCID-gamma mice (NOD.Cg-Prkdcscid Il2rgtm1Wjl/SzJ), both male and female mice were used for experiments and bred in-house. Animals were kept on a 12:12h light-dark cycle and provided with food and water ad libitum in the animal facilities of the Friedrich-Alexander University of Erlangen-Nürnberg. We used 8-12-week-old NOD/SCID-gamma mice in a randomized, non-blinded manner. Male and female mice were distributed equally across the different conditions.
Wild animals	n/a
Reporting on sex	We used 12 male and 12 femal mice.
Field-collected samples	n/a
Ethics oversight	Animal experiments were conducted under licencelicense 55.2.2-2532-2-2150-19 approved by the Regierungspräsidium Unterfranken, Germany.

Note that full information on the approval of the study protocol must also be provided in the manuscript.

Plants

Seed stocks	n/a
Novel plant genotypes	n/a
Authentication	n/a

ChIP-seq

Data deposition

☒ Confirm that both raw and final processed data have been deposited in a public database such as GEO.

☒ Confirm that you have deposited or provided access to graph files (e.g. BED files) for the called peaks.

Data access links<br>*May remain private before publication.*	ChIP-seq data generated for this project have been deposited in the GEO database under accession number (GSE256001 - subseries: GSE255997). Access to unprocessed raw data of primary cells is restricted to protect the privacy and intent of research participants consistent with the informed consent agreements provided by individual research participants. For these samples, we provide processed data.
Files in database submission	GSM8083960 ChIP_PTC_1_ctrl_Input<br>GSM8083961 ChIP_PTC_1_ctrl_H3K27ac<br>GSM8083962 ChIP_PTC_2_ctrl_Input<br>GSM8083963 ChIP_PTC_2_ctrl_H3K27ac<br>GSM8083964 ChIP_PTC_3_ctrl_Input<br>GSM8083965 ChIP_PTC_3_ctrl_H3K27ac<br>GSM8083966 ChIP_ccRCC_1_ctrl_Input<br>GSM8083967 ChIP_ccRCC_1_ctrl_H3K27ac<br>GSM8083968 ChIP_ccRCC_2_ctrl_Input<br>GSM8083969 ChIP_ccRCC_2_ctrl_H3K27ac<br>GSM8083970 ChIP_ccRCC_3_ctrl_Input<br>GSM8083971 ChIP_ccRCC_3_ctrl_H3K27ac<br>GSM8083972 ChIP_U87_DMOG_Input<br>GSM8083973 ChIP_U87_DMOG_H3K27ac<br>GSM8083974 ChIP_U87_DMOG_HIF1_beta
Genome browser session<br>(e.g. UCSC)	n/a

Methodology

Replicates	ChIP-seq in primary tubule and ccRCC cells was conducted in 3 biological replicates. Technical replicates could not be performed due to limited cell availability.<br>HIF-1β ChIP-seq data acquired in U-87 cells were validated by locus-specific qPCR in a second ChIP experiment.
Sequencing depth	ChIP-libraries were sequenced on an Illumina Novaseq 6000 platform to a 2 × 150 paired-end format. The number of total mapped and uniquely mapped reads ranged from 19 Mio to 40 Mio reads.
Antibodies	HIF-1β (NB100-110, Novus Biologicals, Littleton, CO, USA)<br>H3K27ac (ab4729, Abcam, Cambridge, UK)
Peak calling parameters	Peaks were identified applying MACS2 (v2.2.7.1).<br>For peak calling of histone marks the following command line was used: macs2 callpeak -t “Histone_mark_file.bam” -c “Input_file.bam” -f BAMPE -n “sample_name” -B -q 0.01 --outdir “name_of_results_folder”<br>For peak calling of transcription factor binding sites the command line was: macs2 callpeak -t “TF_file.bam” -c “Input_file.bam” -f BAMPE -n “sample_name” --outdir “name_of_results_folder”
Data quality	Significant peaks for transcription factor binding sites were determined at a FDR of 5% (HIF-1β in DMOG-treated U-87 cells: 2254 peaks). Significant peaks for H3K27ac signals were identified at a FDR of 1% and ranged between 20650 and 63021.
Software	Raw sequencing files in fastq format were generated via fastq-dump (2.8.0) and adapter sequences were trimmed using Trim Galore (0.6.6). Quality control was conducted with FastQC (v0.11.8). Reads were mapped to the hg38 reference genome using Burrows-Wheeler Aligner (BWA-mem; 0.7.17-r1188). SAMtools (1.8) removed aligned reads with a Mapping Quality (MAPQ) below 30 and those mapped to ChrM. PCR-duplicates reads were excluded via Picard. We excluded ENCODE blacklisted regions and generated normalized BigWigs for visualization using BamCoverage (3.3.2). Peaks were identified applying MACS2 (v2.2.7.1).
